# Sex Differences in Intestinal Carbohydrate Metabolism Promote Food Intake and Sperm Maturation

**DOI:** 10.1016/j.cell.2019.07.029

**Published:** 2019-08-08

**Authors:** Bruno Hudry, Eva de Goeij, Alessandro Mineo, Pedro Gaspar, Dafni Hadjieconomou, Chris Studd, Joao B. Mokochinski, Holger B. Kramer, Pierre-Yves Plaçais, Thomas Preat, Irene Miguel-Aliaga

**Affiliations:** 1 MRC London Institute of Medical Sciences, Imperial College London, Hammersmith Campus, Du Cane Road, London W12 0NN, UK; 2 Université Côte d’Azur, CNRS, INSERM, iBV, France; 3 Genes and Dynamics of Memory Systems, Brain Plasticity Unit, CNRS, ESPCI Paris, PSL Research University, 10 rue Vauquelin, 75005 Paris, France

**Keywords:** intestine, gonad, testes, gender differences, carbohydrate metabolism, sperm, citrate, organ plasticity, *Drosophila*, sexual dimorphisms

## Abstract

Physiology and metabolism are often sexually dimorphic, but the underlying mechanisms remain incompletely understood. Here, we use the intestine of *Drosophila melanogaster* to investigate how gut-derived signals contribute to sex differences in whole-body physiology. We find that carbohydrate handling is male-biased in a specific portion of the intestine. In contrast to known sexual dimorphisms in invertebrates, the sex differences in intestinal carbohydrate metabolism are extrinsically controlled by the adjacent male gonad, which activates JAK-STAT signaling in enterocytes within this intestinal portion. Sex reversal experiments establish roles for this male-biased intestinal metabolic state in controlling food intake and sperm production through gut-derived citrate. Our work uncovers a male gonad-gut axis coupling diet and sperm production, revealing that metabolic communication across organs is physiologically important. The instructive role of citrate in inter-organ communication might be significant in more biological contexts than previously recognized.

## Highlights

• Intestinal carbohydrate metabolism is male-biased and region-specific
• Testes masculinize gut sugar handling by promoting enterocyte JAK-STAT signaling
• The male intestine secretes citrate to the adjacent testes
• Gut-derived citrate promotes food intake and sperm maturation

## Graphical Abstract

**Figure:**
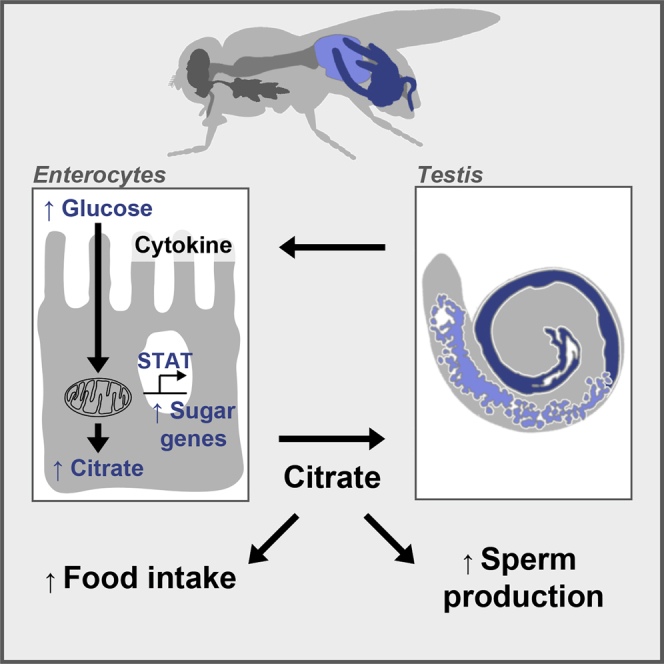

## Teaser

Inter-organ communication couples diet with gamete production. The male gonad promotes sex differences in carbohydrate metabolism within an adjacent intestinal portion via JAK-STAT signalling. In response to this gonadal signal, gut-derived citrate controls food intake and sperm maturation.

## Introduction

Males and females differ in their physiology and disease susceptibility (Link and Reue, 2017, Ober et al., 2008) yet the sex of cells and animals has often been neglected in research, or a single sex (male) is preferentially used (Wald and Wu, 2010). This might have prevented identification of sex differences that could inform clinical studies and therapies. Pressure to consider both sexes in basic and clinical research is revealing that sex differences are extensive, yet relatively underexplored (Clayton and Collins, 2014, Mauvais-Jarvis et al., 2017, Nielsen et al., 2017, Wizemann and Pardue, 2001).

Sex chromosome sensing in *Drosophila melanogaster* activates a splicing cascade that results in expression of the RNA-binding protein Tra^F^ only in females (Boggs et al., 1987), leading to sex-specific splicing of the transcription factors Doublesex (Dsx) and Fruitless (Fru) (Baker and Ridge, 1980, Ryner et al., 1996) in a subset of cells, which sculpt sexually dimorphic anatomical features, reproductive systems, and behavior (Auer and Benton, 2016, Camara et al., 2008, Christiansen et al., 2002, Clough and Oliver, 2012, Dickson, 2008, Villella and Hall, 2008). Although superficially distinct from mammalian mechanisms involving gonadal release of sex hormones, *Drosophila* and mammalian sex differentiation shares common effectors such as the Dmrt/Dsx family of transcription factors (Arnold, 2017, Bellott et al., 2017, Kopp, 2012, Zarkower, 2002). Furthermore, mouse models have revealed a cell-intrinsic contribution of sex chromosome complements to sex differences in body size and adiposity in mammals (Chen et al., 2012, Chen et al., 2013, Link et al., 2017, Zore et al., 2018), and studies in flies have hinted at cell-extrinsic contributions to sex-biased phenotypes (Rideout et al., 2015, Sawala and Gould, 2017, Sieber and Spradling, 2015). Thus, sex differentiation in both insects and mammals appears to be a complex process integrating intrinsic and extrinsic inputs (Ainsworth, 2015, Arnold, 2017).

Like its mammalian counterpart, the adult *Drosophila* digestive tract is a plastic and functionally regionalized organ (Miguel-Aliaga et al., 2018, O’Brien et al., 2011), harboring microbiota and cell types akin to those found in humans, including self-renewing epithelial progenitors, digestive and absorptive enterocytes (ECs), and hormone-secreting enteroendocrine cells (Micchelli and Perrimon, 2006, Miguel-Aliaga et al., 2018, O’Brien et al., 2011, Ohlstein and Spradling, 2006). We recently revealed sex differences in intestinal stem cell proliferation, which are adult-reversible and intrinsic to the stem cells (Hudry et al., 2016). During the course of these experiments, we also observed intestinal sex differences in metabolic gene expression (Hudry et al., 2016), suggesting that sex-biased intestinal metabolism might contribute to sex differences in whole-body physiology.

The intestine communicates with other organs, and peptide hormones are well established mediators (Ameku et al., 2018, Droujinine and Perrimon, 2016, Gribble and Reimann, 2016, Karsenty and Olson, 2016, Scopelliti et al., 2018, Song et al., 2017). However, intermediate products of intracellular, housekeeping metabolic pathways are detected in the circulation, and recent work is revealing that both healthy tissues and tumors can use (and sometimes require) such exogenous, circulating metabolites (Boroughs and DeBerardinis, 2015, Hui et al., 2017, Mills et al., 2018, Jang et al., 2019). Consequently, there is considerable interest in exploring the instructive potential of metabolites in the context of inter-organ signaling (de Castro Fonseca et al., 2016, Yang et al., 2018).

In this manuscript, we uncover bi-directional communication between the male gonad and an adjacent intestinal region. This communication affects both gut and testes function and is mediated by cytokine signaling and the metabolite citrate.

## Results

### Male-Biased and Region-Specific Gene Expression in the Intestine

**Figure S1:**
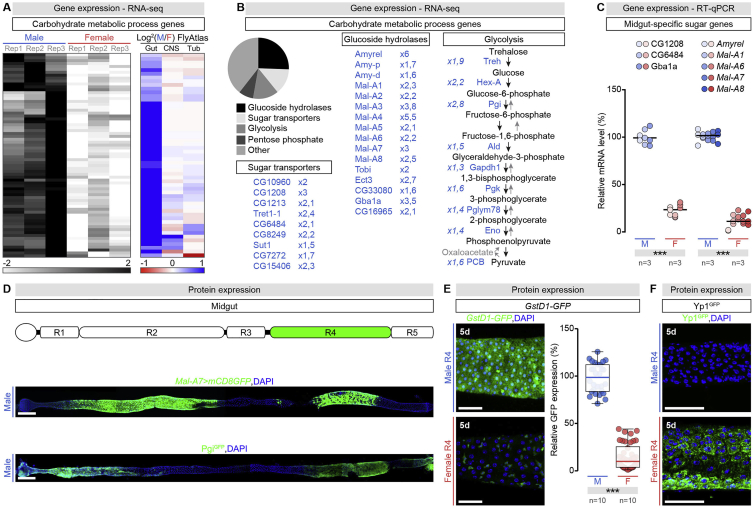
Expression of the Carbohydrate-Handling Machinery Is Male-Biased in a Specific Portion of the Adult Intestine, Related to Figure 1 (A) Heatmap displaying normalized expression abundance for the sugar genes with sexually dimorphic expression in the adult midgut of females and males. Left: using the transcriptional datasets in Hudry et al., 2016). Right: heatmap displaying the normalized ratio of male over female expression of the same genes in the adult midgut, brain (CNS) and Malpighian tubules (Tub), based on FlyAtlas 2 transcriptional data (Leader et al., 2018). See Table S1 for details of genes and their expression abundance. (B) List of the intestinal male-biased sugar genes organized by molecular functions. The specific genes from top to bottom are: *Trehalose transporter 1-1* (*Tret1-1*), *sugar transporter 1* (*sut1*), *Amylase proximal* (*Amy-p*), *Amylase distal* (*Amy-d*), *Maltase A1* (*Mal-A1*), *Maltase A2* (*Mal-A2*), *Maltase A3* (*Mal-A4*), *Maltase A4* (*Mal-A4*), *Maltase A5* (*Mal-A5*), *Maltase A6* (*Mal-A6*), *Maltase A7* (*Mal-A7*), *Maltase A8* (*Mal-A8*), *target of brain insulin* (*tobi*), *Glucocerebrosidase 1a* (*Gba1a*), *Trehalase* (Treh), *Hexokinase-A* (*Hex-A*), *Phosphoglucose isomerase* (*Pgi*), *Aldolase* (*Ald*), *Glyceraldehyde 3 phosphate dehydrogenase 1* (*Gapdh1*), *Phosphoglycerate kinase* (*Pgk*), *Phosphoglyceromutase 78* (*Pglym78*), *Enolase* (*Eno*), *Pyruvate carboxylase* (*PCB*). (C) RT-qPCR expression data for a subset of gut-specific sugar genes (specified at the top of the graph) in male (M) and female (F) control flies. In this and all subsequent figures, expression abundance for each gene was arbitrarily set up at 100% for control males, and percentage of that expression is displayed for the other sex and genotypes. (D) Expression pattern of *Maltase-A7-Gal4* reporter and Pgi^GFP^ protein in whole midguts of adult *Drosophila* (DNA: DAPI, in blue; GFP, in green). The R4 region is highlighted in green in the cartoon above depicting midgut regionalisation. (E) Quantification of *Glutathione S transferase D1-GFP* (*GstD1-GFP*) reporter expression levels in the R4 region of adult male (M) and female (F) midguts. Representative images are shown (DNA is labeled with DAPI in blue; *GstD1-GFP* is visualized in green with GFP). In this panel, GFP expression abundance was set up at 100% for control males, and expression in females is displayed as percentage of male expression. (F) Expression of Yolk protein 1^GFP^ (Yp1^GFP^) in the R4 region of adult male (M) and female (F) midguts. Representative images are shown (DNA is labeled with DAPI in blue; Yp1 is visualized in green with GFP). n denotes the number of groups of flies analyzed for each genotype (each group consisting of 20 flies) in (C), and the number of midguts in (E). Scale bars, 50 μm in all images except for (D) 200 μm. Asterisks highlighting significant comparisons across sexes are displayed in gray boxes at the bottom of the graphs. In this and all subsequent figures, data is presented as boxplots with all data points shown, p values from Mann-Whitney-Wilcoxon test (non-significant (ns): p > 0.05; ^∗^: 0.05 > p > 0.01; ^∗∗^: 0.01 > p > 0.001; ^∗∗∗^p < 0.001). See Table S4 for a list of full genotypes. See also Table S1.

**Figure 1:**
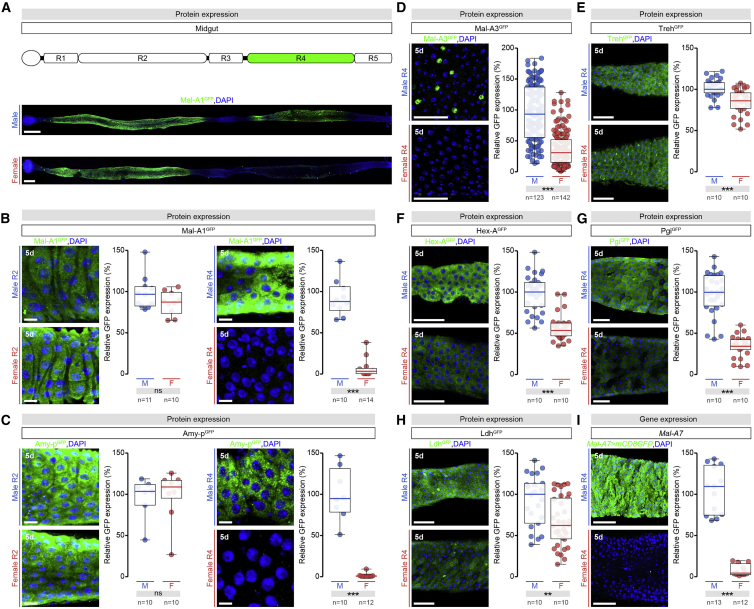
Expression of Carbohydrate Metabolism Proteins Is Male-Biased in a Specific Portion of the Adult Intestine (A) Expression pattern of Maltase A1 (Mal-A^GFP^) protein in whole midguts of adult *Drosophila* (DNA labeled with DAPI, blue; Mal-A1^GFP^, green). (B and C) Quantifications of Mal-A1^GFP^ (B) and Amylase proximal (Amy-p^GFP^) (C) protein levels in R2 (left) and R4 (right) regions of adult male (M) and female (F) midguts. Representative images are shown (DAPI, blue; protein, GFP in green). For plots (including subsequent graphs), expression levels were set at 100% for control males, expression in females are displayed as a percentage of male expression. (D–H) Representative images (DAPI, blue; protein, GFP in green) and quantifications of Mal-A3^GFP^ in enteroendocrine cells (D), Trehalase (Treh^GFP^) (E), Hexokinase A (Hex-A^GFP^) (F), Phosphoglucose isomerase (Pgi^GFP^) (G), and Lactate dehydrogenase (Ldh^GFP^) (H) expression in R4 region of adult male and female midguts. (I) Representative images (DAPI, blue; *Mal-A7 > mCD8GFP,* green) and quantifications of *Mal-A7-Gal4* expression levels in R4 region of adult male and female midguts. Data combined from at least two independent experiments. n = midgut number per genotype, except in (D), where n = cell number. Scale bars, 50 μm in all images except for 200 μm in (A) and 10 μm in (B), (C), and (I). Asterisks highlighting significant comparisons across sexes displayed in gray boxes at bottom of graphs. In this and subsequent figures, data shown in boxplots with all data points shown, p values from Mann-Whitney-Wilcoxon test (non-significant (ns): p > 0.05; ^∗^0.05 > p > 0.01; ^∗∗^0.01 > p > 0.001; ^∗∗∗^p < 0.001). See Table S4 for a list of full genotypes. See also Figure S1.

Adult virgin flies show male-biased expression of genes with putative functions in carbohydrate transport and utilization (Figure S1A; Table S1) (Hudry et al., 2016), including digestive enzymes (Figure S1B). This sexual dimorphism is predominantly confined to the midgut (Figure S1A; Table S1) (Leader et al., 2018). We validated male-biased expression for a subset of genes coding for carbohydrate handling (breakdown, transport, or utilization) proteins via reverse transcription-quantitative polymerase chain reaction (RT-qPCR); we selected genes with midgut-specific expression so that RT-qPCR profiling could be performed on RNA from whole, adult flies (Figure S1C). To analyze this sexual dimorphism, we engineered protein reporters by tagging endogenous proteins representative of various sugar-handling processes with green fluorescent protein (GFP) (see STAR Methods), including: Phosphoglucose isomerase (Pgi), Maltase-A3 (Mal-A3), and Amylase proximal (Amy-p). Immunohistochemical analyses of these protein reporters, a transcriptional reporter for *Maltase-A7* (*Mal-A7*) (see STAR Methods) and publicly available protein reporters for other enzymes (Maltase A1 [Mal-A1], Trehalase [Treh], Hexokinase A [Hex-A] and Lactate dehydrogenase [Ldh]) confirmed the sexual dimorphism at the protein level and revealed that it was predominantly confined to the intestinal epithelium (Figure 1A–1I). The epithelial cell types contributing to this expression differed depending on whether the protein was involved in sugar breakdown, transport, or utilization, but invariably included the digestive and absorptive ECs (with one exception, Mal-A3, expressed exclusively in enteroendocrine cells) (Figure 1D).

We observed that sexually dimorphic expression was spatially restricted to the posterior R4 region of the adult midgut (Buchon et al., 2013), even when the transcripts or proteins were expressed in other intestinal portions (see Figures 1A and S1D for Mal-A1, *Mal-A7,* and Pgi; quantification is shown in Figures 1B, 1C, and 1I for Mal-A1, Amy-p and *Mal-A7*, respectively). Sexual dimorphism in the R4 region was not restricted to carbohydrate metabolism genes; it also included oxidative stress response genes such as *Glutathione S transferase D1* (*GstD1*) (Figure S1E) and genes with female-biased expression such as Yolk protein 1 (Yp1) (Figure S1F).

Thus, the proteins handling sugars in the adult gut are male-biased, and this intestinal sexual dimorphism is spatially confined to the posterior R4 midgut region.

### Sex Differences in Sugar Gene Expression Are Independent of Gut Cell Sex

**Figure 2:**
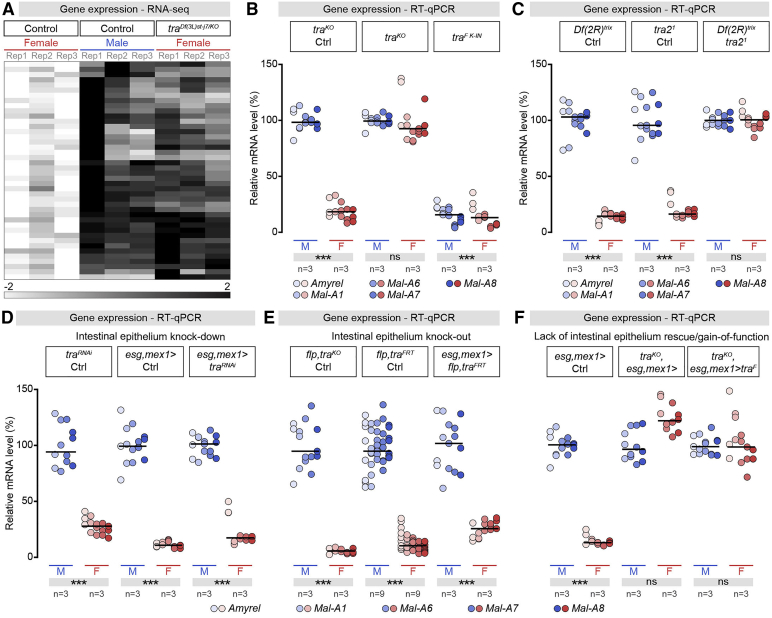
Sex Differences in Intestinal Sugar Gene Expression Are Independent of Gut Cell Sex (A) Heatmaps displaying normalized expression abundance for sugar genes with *transformer* (*tra*)-dependent sexually dimorphic expression in adult midgut of females, males, and whole-body *tra* null mutant “females” (in Table S1 are genes and expression abundance). (B and C) RT-qPCR expression data for a subset of sugar genes with midgut-specific expression (Leader et al., 2018) (gene names at bottom of graphs) in male (M) and female (F) control flies, flies with whole-body *tra* knockout (*tra*^*KO*^) or flies with whole-body *tra*^*F*^ knockin gain-of-function (*tra*^*F K-IN*^) (B) and flies harboring a whole-body *tra2* null mutation (C). In these and subsequent graphs, expression abundance for each gene was arbitrarily set at 100% for control males, and the percentage of that expression is displayed for the other sex and genotypes. (D–F) RT-qPCR expression data for the same set of sugar genes in flies in which *tra* was downregulated (D) (*escargot, midgut expression 1* [esg, mex1] > *tra*^*RNAi*^), knocked out (E) (*esg, mex1 > flp,tra*^*F*^) or re-introduced (F) in a *tra* whole-body mutant (*tra*^*KO*^, *esg, mex1 > tra*^*F*^) in intestinal progenitors and ECs in relation to controls. In all images, n = number of fly groups analyzed per genotype (each group, 20 flies). Asterisks highlighting significant comparisons across sexes are displayed in gray boxes at bottom of graphs. See Table S4 for a list of full genotypes. See also Figures S2 and S3 and Table S1.

**Figure S2:**
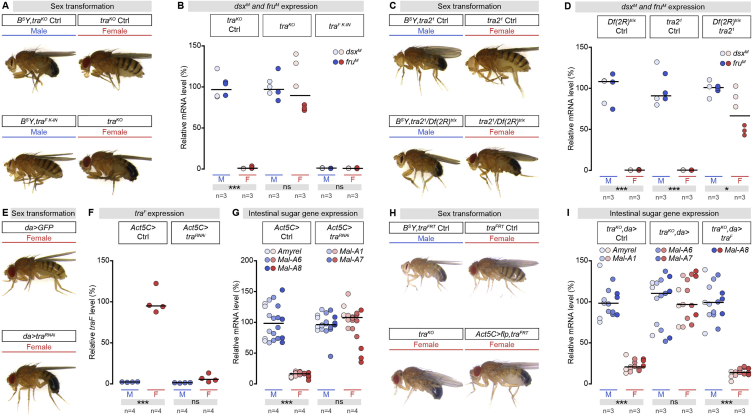
Functional Validation of *transformer* (*tra*) Knockout, Knockin, Gain-of-Function and RNAi Lines, Related to Figure 2 (A) Sex transformations induced by whole-body *tra* knockout (*tra*^*KO*^) and whole-body *tra*^*F*^ knockin gain-of-function (*tra*^*F K-IN*^). Female mutants lacking *tra* display masculinised appearance and are referred to as “pseudomales.” Male expressing *tra* display a feminised appearance and are referred to as “pseudofemales.” (B) RT-qPCR expression data for the male-specific *doublesex* (*dsx*) and *fruitless* (*fru*) transcripts in male (M) and female (F) control flies, flies with whole-body *tra* knockout (*tra*^*KO*^) or flies with whole-body *tra*^*F*^ knockin gain-of-function (*tra*^*F K-IN*^) relative to controls. (C) Sex transformations induced by whole-body *transformer 2* (*tra2*) null mutations (*tra2*^*1*^*/Df(2R)*^*trix*^). Female mutants lacking *tra2* display masculinised pseudomale appearance. (D) RT-qPCR expression data for the male-specific *dsx* and *fru* transcripts in male (M) and female (F) control flies, and in flies harboring a whole-body *tra2* null mutation relative to controls. (E) Sex transformations induced by whole-body *tra* knockdown (*daughterless* (*da) > tra*^*RNAi*^). Females with *da-Gal4*-driven *tra* knockdown display masculinised pseudomale appearance. (F) RT-qPCR expression data for the female-specific *tra* transcript in male (M) and female (F) control flies and flies following whole-body *tra* knockdown (*Actin 5C (Act5C) > tra*^*RNAi*^). (G) RT-qPCR expression data for a set of midgut-specific sugar genes (indicated at the top of the graph) in flies with whole-body *tra* knockdown (*Act5C > tra*^*RNAi*^). *Act5C-Gal4*-driven *tra* knockdown masculinises the intestinal sugar gene expression of females. (H) Sex transformations induced by *Act5C-Gal4* driven *tra* knockout (*Act5C > flippase (flp),tra*^*FRT*^). Females with *Act5C-Gal4*-driven *tra* knockout display masculinised pseudomale appearance. (I) RT-qPCR expression data for a set of midgut-specific sugar genes (indicated at the top of the graph) in flies with whole-body *tra* knockout (*tra*^*KO*^*,da >* ) and with whole-body *tra* knockout rescued by ubiquitous *tra*^*F*^ expression (*tra*^*KO*^*,da > tra*^*F*^). Ubiquitous *tra*^*F*^ re-introduction in *tra* whole-body mutants (*tra*^*KO*^*,da > tra*^*F*^) restores the feminised intestinal sugar gene expression of whole body *tra* mutant females (*tra*^*KO*^*,da >* ) to wild-type female-like levels (*tra*^*KO*^*,da >* control). In all panels, n denotes the number of group of flies analyzed for each genotype (each group consisting of 20 flies). Asterisks highlighting significant comparisons across sexes are displayed in gray boxes at the bottom of the graphs. See Table S4 for a list of full genotypes.

To explore how male-biased intestinal sugar gene expression arises, we used RNA-seq transcriptional analysis, which revealed upregulation of sugar genes in “masculinized” female flies lacking the female sex determinant *tra* (Figure 2A; Table S1). We confirmed that their expression is controlled by Tra^F^ and its binding partner Transformer 2 (Tra2) (Amrein et al., 1988, Fujihara et al., 1978, Goralski et al., 1989) by assessing the effect of whole-body *tra* and *tra2* mutation (masculinization) or *tra*^*F*^ mis-expression (feminization) on the subset of gut-specific, male-biased sugar genes (Figure 2A–2C). We generated a *tra* allele (*tra*^*FRT*^) that allows whole-body or cell-type-specific *tra* deletion and a *tra*^*F K-IN*^ knockin allele that constitutively feminizes males. This allele fully rescues *tra*-null mutant females (unlike *UAS-tra*^*F*^), including their fertility (see STAR Methods and Figures S2A–S2D). Both genetic manipulations abrogated the sex bias in sugar gene expression; *tra*/*tra-2* mutation did so by upregulating the expression of the sugar genes in female (masculinized) flies (Figures 2A–2C and S2I), whereas ectopic *tra*^*F*^ reduced their expression in male (feminized) flies to amounts comparable to those detected in female guts (Figure 2B).

**Figure S3:**
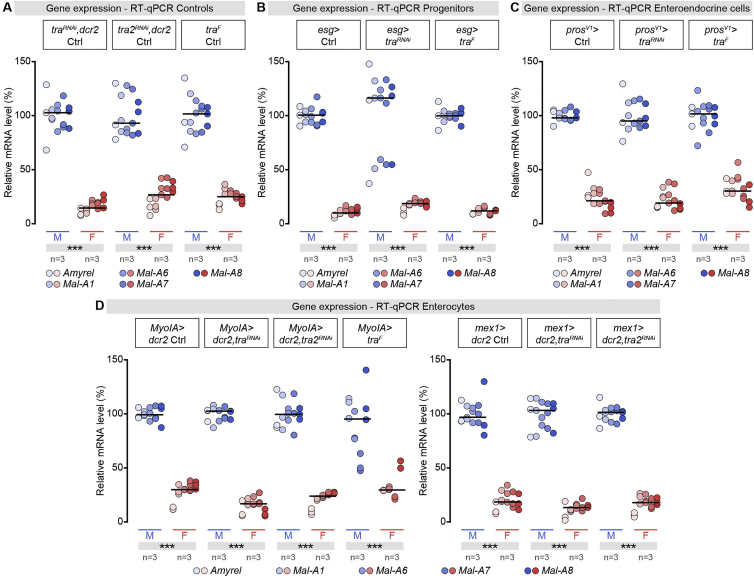
The Sex Differences in Intestinal Sugar Gene Expression Are Independent of the Genetic Sex of Gut Cells, Related to Figure 2 (A) RT-qPCR expression analysis of midgut-specific sugar genes (indicated at the bottom of the graph) in male (M) and female (F) flies of different *UAS* controls. RT-qPCR expression data for the same set of sugar genes in male (M) and female (F) flies with *transformer*^*F*^ knockdown or mis-expression specifically in intestinal progenitors (B, *esgarcot (esg) >* ), enteroendocrine cells (C, *prospero* (*pros*^*V1*^*)>*) and enterocytes (D, *Myosin 31DF* (*MyoIA) >* or *midgut expression 1 (mex1) >* ). None of these manipulations affected the sexual dimorphism in intestinal sugar gene expression. In all panels, n denotes the number of group of flies analyzed for each genotype (each group consisting of 20 flies). Asterisks highlighting significant comparisons across sexes are displayed in gray boxes at the bottom of the graphs. See Table S4 for a list of full genotypes.

We expected that Tra^F^ would control sex differences in intestinal sugar genes intrinsically from the intestinal epithelium itself, like the sex differences in intestinal stem cell proliferation (Hudry et al., 2016). However, the sex-biased intestinal sugar gene expression is *tra2*-dependent, unlike intestinal stem cell proliferation, suggesting that a different mechanism is involved. To investigate this mechanism, we removed Tra^F^/Tra2 function in specific cell types or tissues using *tra* and *tra2* knockdown (KD) lines and the *tra* allele that allows its cell-type-specific deletion (Figures S2E–S2I). Both *tra* and *tra2* downregulation and *tra* mutation failed to affect male bias in intestinal sugar gene expression when confined to the intestinal epithelium (Figures 2D, 2E, and S3A–S3D). Attempts to rescue the “masculinization” of intestinal sugar gene expression in *tra* mutant females by re-instating *tra*^*F*^ expression in the intestinal epithelium were also unsuccessful (Figure 2F). Similarly, although forced expression of *tra*^*F*^ in all fly tissues “feminized” intestinal sugar gene expression in genotypically male flies (Figure S2I), we failed to observe such “feminization” when mis-expression was confined to the different intestinal epithelial cell types (Figures S3A–S3D).

Thus, two distinct *tra*-dependent mechanisms impart sex differences to the intestinal epithelium; the intrinsic (and *tra2*-independent) sexual identity of adult intestinal progenitors controls their female-biased proliferation (Hudry et al., 2016), whereas a gut-extrinsic, *tra2*-dependent mechanism controls the male bias in intestinal sugar gene expression.

### The Male Gonad Extrinsically Controls Region-Specific Intestinal Sugar Gene Expression

**Figure S4:**
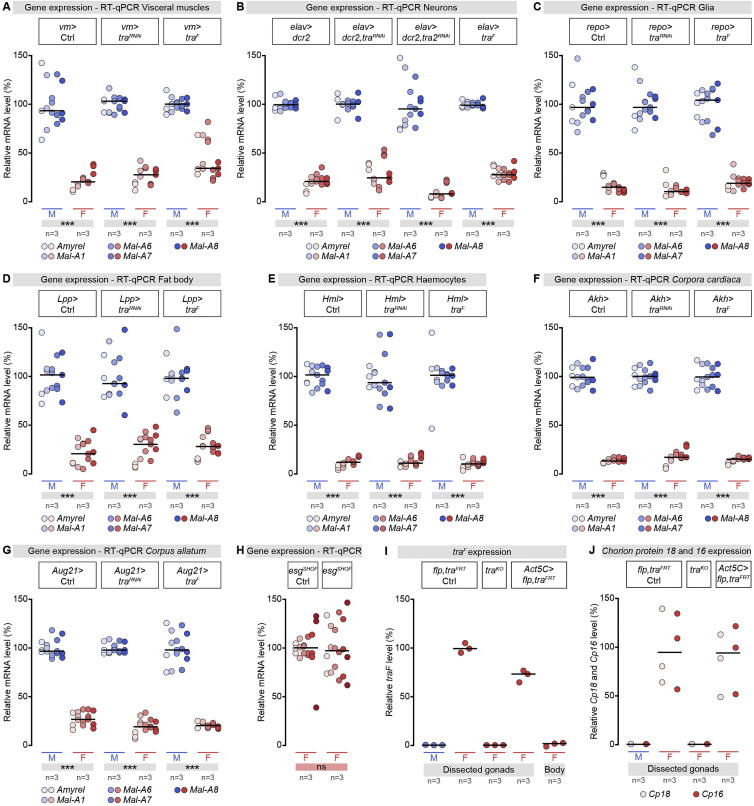
Tissue-Specific Screen to Identify the Cell Types and/or Organs Controlling Intestinal Sex Differences in Sugar Gene Expression, Related to Figure 3. (A–G) RT-qPCR expression analysis of midgut-specific sugar genes (indicated at the bottom of the graph) in male (M) and female (F) flies following *transformer*^*F*^ (*tra*^F^) knockdown or mis-expression specifically in visceral muscles (A, *vm >* ), neurons (B, *embryonic lethal abnormal vision* (*elav) >* ), glial cells (C, *reversed polarity (repo) >* ), fat body cells (D, *apolipophorin* (*Lpp) >* ), hemocytes (E, *Hemolectin* (*Hml) >* ), and secretory glands such as the *corpora cardiaca* (F, *Adipokinetic hormone* (*Akh) >* ) and *corpus allatum* (G, *Aug21 >* ). None of these manipulations affected the sexual dimorphism in intestinal sugar gene expression. (H) RT-qPCR expression analysis of the same midgut-specific sugar genes in *shutoff* (*esg*^*SHOF*^) females relative to control females (control for Figure 3E). (I) RT-qPCR expression data for the female-specific *tra* transcript in male (M) and female (F) dissected gonads of controls, whole-body *tra* knockout (*tra*^*KO*^) and *Actin 5C (Act5C)-Gal4*-driven *tra* knockout (*Act5C > flippase (flp),tra*^*FRT*^), and rest of body of *Act5C-Gal4*-driven *tra* knockout. As expected from their anatomical masculinisation (Figure 3B), *tra* excision leads to loss of *tra*^*F*^ expression in the body (minus gonads) of *Act5C-Gal4*-driven *tra* knockouts, but *tra*^*F*^ expression is retained in their ovaries. This is in contrast to *tra*^*KO*^ flies, in which *tra*^*F*^ expression is also lost in gonads. (J) RT-qPCR expression data for the *Chorion protein 18* and *16* transcripts, used as transcriptional readouts of ovarian differentiation (Griffin-Shea et al., 1982), in male (M) and female (F) dissected gonads of controls, whole-body *tra* knockout (*tra*^*KO*^) and *Act5C-Gal4*-driven *tra* knockout (*Act5C > flp,tra*^*FRT*^). As expected from their anatomical features (Figure 3B), these transcripts are absent from *tra*^*KO*^ masculinised females, but are retained in *Act5C-Gal4*-driven *tra* knockout females, in which *tra* expression and female identity have been spared in the gonad. In all panels, n denotes the number of group of flies analyzed for each genotype (each group containing 20 flies). Asterisks highlighting significant comparisons across sexes are displayed in gray boxes at the bottom of the graphs. See Table S4 for a list of full genotypes.

To analyze the extrinsic factors influencing intestinal sugar gene expression, we “feminized” or “masculinized” specific cell types or tissues by confining *tra*/*tra-2* KD or *tra*^*F*^ mis-expression via tissue-specific driver lines. Targeting visceral muscles (Figure S4A), neurons (Figure S4B), glia (Figure S4C), fat body (with liver and adipose tissue-like functions) (Figure S4D), immune cells (hemocytes) (Figure S4E), or secretory glands such as the *corpora cardiaca* and *corpus allatum* (Figures S4F and S4G, respectively) all failed to affect male bias in intestinal sugar gene expression, suggesting that these tissues were unlikely to be the source of a sex-biased signal.

**Figure 3:**
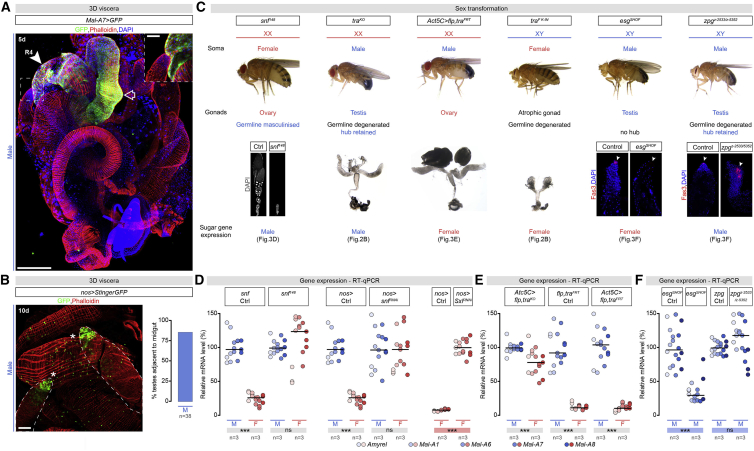
Male Gonad Is Adjacent to Midgut R4 Region and Extrinsically Controls Region-Specific Intestinal Sugar Gene Expression (A) Immunostaining of abdominal internal organs in their intact three-dimensional organization (DAPI, blue; *Maltase-A7 (MalA7)>GFP*, green; muscles, Phalloidin/red). R4 intestinal region (white arrow and inset) is adjacent to apical tip of testes (scale bar, 200 μm except in inset, 50 μm). R2, hollow arrow. (B) Quantification of intestine-testes proximity in intact male abdomens. A representative image is shown. Apical tips of testes are highlighted with white asterisks and visualized with GFP/green (*nanos* [nos] *> UAS-StingerGFP*); muscles with Phalloidin/red. (C) For each boxed genotype, a summary of sex chromosome complements (XY or XX), sexual phenotype of the soma (whole fly images), presence or state of gonads (representative images below each description) and intestinal sugar gene expression are displayed. Genotypes, left to right: female-sterile *snf*^*148*^ female flies, (confocal image shows wild-type ovariole, left, with egg chambers spanning the fourteen stages of oogenesis; *snf* mutant ovariole, right, lacking differentiating egg chambers); whole-body *tra* knockout (*tra*^*KO*^) “masculinized” females; *Actin5C-Gal4*-driven *tra* knockout (*Act5C > flippase (flp),tra*^*FRT*^) females; whole-body *transformer*^*F*^ (*tra*^*F K-IN*^) knockin gain-of-function feminized males; sterile*shutoff* (*esg*^*SHOF*^) male flies (hub cells labeled with Fasciclin 3 [Fas3] in red; DAPI, blue); and sterile *zero population growth* (*zpg*)^*z-2533/z-5352*^ male flies (intact hub cells, labeled with Fasciclin 3 [Fas3] in red, DAPI, blue) (Smendziuk et al., 2015). (D) RT-qPCR expression analysis of midgut-specific sugar genes in males (M) and females (F) with germline-specific *sans fille* (*snf*) mutation (*snf*^*148*^), KD (*nos > snf*^*RNAi*^) or germline-specific *Sex lethal* (*Sxl)* KD (*nos > Sxl*^*RNAi*^) in relation to relevant controls. (E) RT-qPCR expression analysis of midgut-specific sugar genes in flies with *Act5C-Gal4* driven *tra* KD (*Act5C > flp,tra*^*FRT*^) in relation to controls. (F) RT-qPCR expression analysis of midgut-specific sugar genes in sterile male flies lacking hub and germline stem cells (*esg*^*SHOF*^), and in sterile males lacking germline but with intact hub (*zpg*^*z-2533/z-5352*^*)*. In all graphs, n = number of fly groups analyzed per genotype (each group, 20 flies), except in (B), where n = number of viscera analyzed. Asterisks highlighting significant comparisons across sexes are displayed in gray boxes at bottom of graphs; those highlighting significant comparisons within female and male datasets are displayed in red and blue boxes, respectively. See Table S4 for a list of full genotypes.

Given that our previous findings ruled out the intestinal epithelium as a source of an extrinsic factor(s), we examined the spatial restriction of the intestinal sexual dimorphism in sugar gene expression in more detail—in particular, its three-dimensional arrangement inside the male body cavity. Immunohistochemical analysis of the internal organs in their intact arrangement (see STAR Methods) revealed close proximity between the gut region with male-biased sugar gene expression and the apical tip of the testes (Figures 3A and 3B).

We hypothesized that gonadal sex might control intestinal sugar gene expression and generated a series of flies in which we uncoupled gonadal from somatic sex. Masculinization of female gonads in otherwise female flies resulted in male-like intestinal sugar gene expression. This was the case in *sans fille* (*snf)* mutant female flies, or in female flies with germline-specific *Sex lethal (Sxl)* or *snf* KDs, which resulted in de-repression of testis genes in the “female” gonad (Casper and Van Doren, 2009, Chau et al., 2009, Chau et al., 2012, Shapiro-Kulnane et al., 2015, Primus et al., 2019) (Figures 3C and 3D). Comparison of two *tra* mutations with different effects on the gonad pointed to a requirement for the male somatic gonad rather than the germline itself. *tra*^*KO*^ mutant “females,” which have masculinized somatic tissues and pseudo-testis that develop as testis but lack male germ cells (Yang et al., 2012), had high, male-like intestinal sugar gene expression (Figures 2B and 3C). By contrast, low, female-like intestinal sugar gene expression was observed in *tra* mutants generated by ubiquitous excision of the excisable *tra* allele (Figure 3E). Like *tra*^*KO*^ mutants, these mutant “female” flies have masculinized tissues but, unlike *tra*^*KO*^ mutants, they develop ovaries (Figures 3C, S4I, and S4J). These two mutants indicate that intestinal sugar gene expression is dependent of the sex of the gonad rather than the sex of the rest of the body. We observed female-like intestinal sugar gene expression in feminized *tra*^*F*^ knockin “males” in which all tissues are feminized but have atrophic gonads, also consistent with a male gonad requirement (rather than, for example, a repressive signal emanating from the female gonad) (Evans and Cline, 2007, Yang et al., 2012) (Figures 2B and 3C).

To demonstrate a contribution of the male somatic gonad more directly, we used *shutoff* (*esg*^*SHOF*^) mutant male flies lacking a functional testis (Voog et al., 2014) (Figure 3C). Absence of a male gonad in these otherwise male flies resulted in low, “feminized” intestinal sugar gene expression (Figures 3F and S4H). To confirm the involvement of the male somatic gonad (as opposed to the male germline), we used *zero population growth* (*zpg*) mutants, which lack the male germline but have an intact somatic hub (Gilboa et al., 2003, Smendziuk et al., 2015, Tazuke et al., 2002). Unlike *esg*^*SHOF*^ males, these males still displayed a male-like pattern of intestinal sugar gene expression (Figures 3C and 3F).

Overall, these experiments indicate that gonadal sex controls sex differences in intestinal sugar gene expression and point to a signal derived from the male somatic gonad as the molecular mediator.

### The Male Gonad Promotes Intestinal Sugar Gene Expression by Activating JAK-STAT

**Figure S5:**
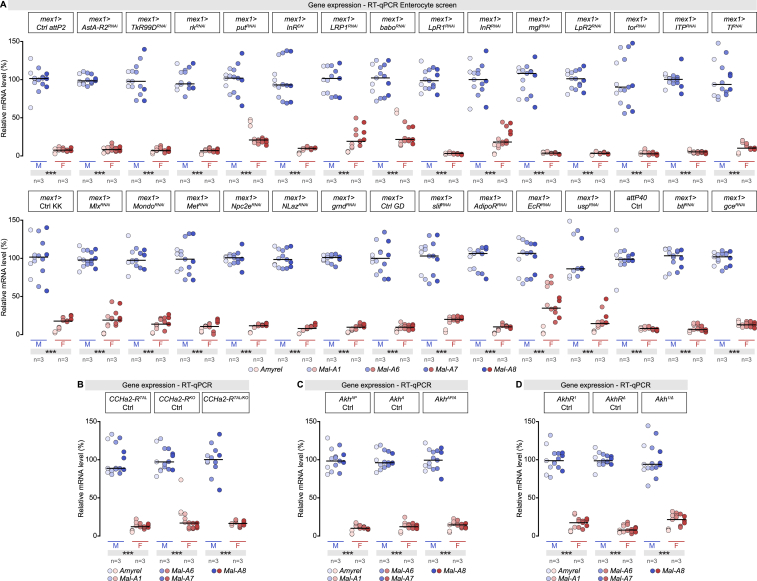
Enterocyte-Specific Knockdown Screen to Identify the Signaling Pathway Driving Intestinal Sex Differences in Sugar Gene Expression, Related to Figure 4 (A) RT-qPCR expression analysis of midgut-specific sugar genes (indicated at the bottom of the graph) in male (M) and female (F) flies following enterocyte-specific knockdown of signal transduction components. The specific genes targeted from top to bottom are: *Allatostatin A receptor 2* (*Asta-R2*), *Tachykinin-like receptor at 99D* (*TkR99D*), *rickets* (*rk*), *punt* (*put*), *Insulin-like receptor* (*InR*), *LDL receptor protein 1* (*LRP1*), *baboon* (*babo*), *Lipophorin receptor 1* (*LpR1*), *Megalin* (*mgl*), *Lipophorin receptor 2* (*LpR2*), *torso* (*tor*), *Ion transport peptide* (*ITP*), *Toll* (*Tl*), *bigmax* (*Mlx*), *Methoprene-tolerant* (*Met*), *Niemann-Pick type C-2e* (*Npc2e*), *Neural Lazarillo* (*Nlaz*), *grindelwald* (*grnd*), *slimfast* (*slif*), *Adiponectin receptor* (*AdipoR*), *Ecdysone receptor* (*EcR*), *ultraspiracle* (*usp*), *breathless* (*btl*), *germ cell-expressed bHLH-PAS* (*gce*). (B–D) RT-qPCR expression analysis of the same midgut-specific sugar genes in male (M) and female (F) flies with *CCHamide-2 receptor* (B, *CCHa2-R*^*TAL/KO*^), *Adipokinetic hormone* (C, *Akh*^*AP/A*^), and *Adipokinetic hormone receptor* (*D*, *AkhR*^*1/Δ*^) null mutations. None of these genetic manipulations affected the sexual dimorphism in intestinal sugar gene expression. In all panels, n denotes the number of group of flies analyzed for each genotype (each group contains 20 flies). Asterisks highlighting significant comparisons across sexes are displayed in gray boxes at the bottom of the graphs. See Table S4 for a list of full genotypes.

**Figure 4:**
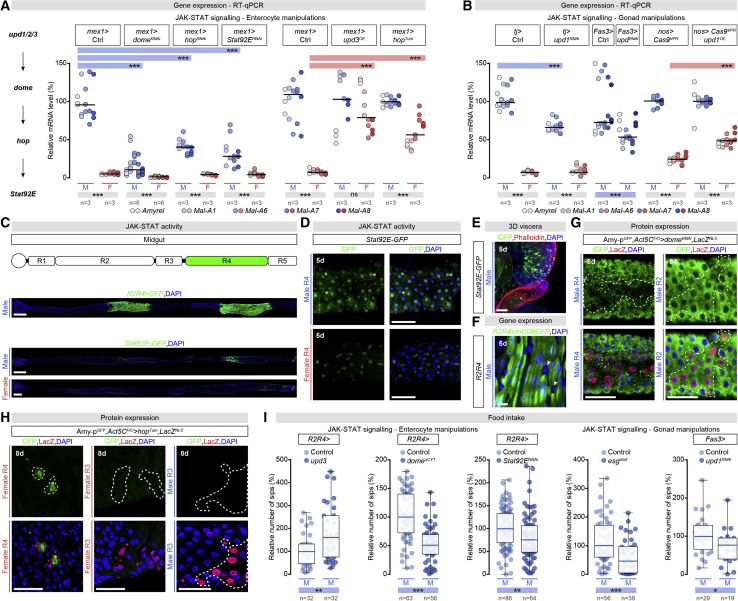
Gonadal Activation of Intestinal JAK/STAT Signaling Promotes Intestinal Sugar Gene Expression in Male Enterocytes of the R4 Region (A) RT-qPCR expression analysis of midgut-specific sugar genes in males (M) and females (F) following EC-specific KD of JAK-STAT receptor *domeless (dome)* (*mex1 > dome*^*RNAi*^), downstream signaling transducers *hopscotch (hop)* (*mex1 > hop*^*RNAi*^), and *Signal-transducer and activator of transcription protein at 92E (Stat92E)* (*mex1 > Stat92E*^*RNAi*^), or EC-specific mis-expression of constitutively active Hop (*mex1 > hop*^*Tum*^), and JAK-STAT ligand *(unpaired 3) upd3 (mex1 > upd3*^*OE*^). (B) RT-qPCR expression analysis of midgut-specific sugar genes after testis-specific downregulation of *unpaired 1* (*upd1)* (*traffic jam (tj) > upd1*^*RNAi*^ and *fascilin 3 (fas3 ) > upd1*^*RNAi*^), or ectopic expression of *upd1* from female germline (*nos > Cas9*^*VPR*^*upd1*^*OE*^), compared with both relevant controls and flies of the opposite sex with an identical genetic manipulation. (C) Representative midgut expression of *Stat92E-GFP* and *R2R4-Gal4* reporters (DAPI, blue; reporter-driven GFP, green). (D) Higher magnification images of *Stat92E-GFP* expression (green) in the R4 region of male and female midguts. ECs are identified by larger DAPI-positive nuclei, blue. (E) Immunostaining of male midgut and testes in intact three-dimensional arrangement (DNA: DAPI, blue; *Stat92E-GFP*: GFP, green; Actin: Phalloidin, red). (F) *R2R4-Gal4* reporter is expressed only in ECs (with larger, endoreplicating nuclei, DAPI, blue), not in intestinal progenitors or enteroendocrine cells (small DAPI-positive nuclei, white arrowheads) (*R2R4 > mCD8GFP*, GFP, green). (G and H) Expression of Amy-p (green, Amy-p^GFP^) after clonal KD of JAK-STAT receptor *dome (dome*^*RNAi*^*)* (G) or clonal production of constitutively active Hop protein (*hop*^*Tum*^) (H) (DNA: blue, DAPI; anti-beta galactosidase, red, staining LacZ-positive cells inside the clone in which *dome*^*RNAi*^ or *hop*^*Tum*^ expression has been induced). (I) Food intake quantifications based on FlyPAD-monitored sips per male fly after R2 and R4 EC-specific manipulations of JAK-SAT signaling (left) or interference with gonadal JAK-STAT ligand production (right). Manipulations, left to right: mis-expression of JAK-STAT ligand Upd3 *(mex1 > upd3*^*OE*^*)*, expression of dominant-negative JAK-STAT receptor (*mex1 > dome*^*ΔCYT*^), R2 and R4 EC-specific *Stat92E* KD (*mex1 > Stat92E*^*RNAi*^), loss of testis hub cells (*esg*^*SHOF*^), and hub-cell-specific *upd1* KD (*fas3 > upd1*^*RNAi*^). For each manipulation, median number of sips was arbitrarily set at 100% for control males and the percentage of that expression was displayed for other genotypes. n = number of fly groups analyzed per genotype in (A) and (B) (each group, 20 flies), or fly numbers monitored via FlyPAD in (I).Asterisks highlighting significant comparisons across sexes are displayed in gray boxes, those highlighting significant comparisons within same-sex datasets are displayed in blue boxes (males) and red boxes for females. Scale bars, 50 μm in all images except for 200 μm in (C) and 10 μm in (I). See Figure S4 for a list of full genotypes. See also Figure S5.

We hypothesized that the male gonad activates a signaling pathway in gut cells in a sexually dimorphic manner, leading to male-biased expression of sugar genes. To identify this pathway, we conducted a genetic screen by knocking down signal transduction components in ECs, including major hormonal pathways (e.g., juvenile hormone and ecdysone) (Droujinine and Perrimon, 2016), pathways with a sexually dimorphic signature in our transcriptional analysis (fibroblast growth factor [FGF] signaling, peptidergic signaling by Allatostatin A, Bursicon, and Tachykinin) (Hudry et al., 2016), and/or pathways that modulate carbohydrate metabolism (e.g., insulin, Mondo, Bigmax (Mlx), Dawdle) (Chng et al., 2014, Mattila and Hietakangas, 2017). RNAi was used to KD expression in ECs (Figure S5A)—or amorphic mutants were used when available (Figures S5B–S5D). Only interference with the Janus kinase/signal transducers and activators of transcription (JAK-STAT) signaling pathway (Hombría and Brown, 2002) reduced male bias in intestinal sugar gene expression (Figure 4A).

Consistent with male-biased activation of the JAK-STAT pathways in ECs, a Stat signaling reporter (*Stat92E-GFP*) (Bach et al., 2007) displayed broader epithelial expression in the R4 midgut region of males than in females (Figures 4C and 4D), especially in the gut portion in closest proximity to the testis hub (Figure 4E). A candidate ligand that could activate the JAK-STAT pathway in ECs was the cytokine Unpaired 1 (Upd1) (Hombría and Brown, 2002, Rajan and Perrimon, 2012, Sáinz et al., 2015). Upd1 is produced by the testis hub and promotes self-renewal of male somatic cyst stem cells and germ stem cell adhesion (Greenspan et al., 2015, Kiger et al., 2001, Leatherman and Dinardo, 2010, Tulina and Matunis, 2001). Downregulation of *upd1* from testis somatic cells reduced intestinal sugar expression in male guts (Figure 4B), although to a lesser extent than interfering with JAK-STAT receptor or downstream signaling from ECs, suggesting incomplete ligand downregulation and/or partial ligand redundancy.

Masculinization of intestinal sugar gene expression has been observed in mutant females with “masculinized” tumorous ovaries, such as *snf* or *nanos (nos) > Sxl-RNAi* females in which the transformed ovaries ectopically activate JAK-STAT ligands and pathway components (Figure 3C) (Shapiro-Kulnane et al., 2015). To further test whether ectopic JAK-STAT signaling affects inter-organ sex differences in females, we: (1) ectopically expressed Upd1 from a wild-type female gonad by using *nos-Gal4* and *Cas9-*VP64-p65-Rta fusion (Cas9^VPR^); and (2) ectopically activated the JAK-STAT pathway in female ECs by expressing a constitutively active Hopscotch (Hop) (*UAS-hop*^*Tum*^) or the JAK-STAT ligand Unpaired 3 (Upd3) from *midgut expression 1 (mex1)-Gal4*. In both cases, intestinal sugar gene expression was upregulated in female guts (Figures 4A and 4B).

To explore how JAK-STAT signaling conferred male identity on ECs, as well as its range of action, we induced flip-out clones (Harrison and Perrimon, 1993) in adult flies in which we either downregulated the JAK-STAT receptor *domeless (dome)* in males, or ectopically activated JAK-STAT signaling in females. Clones with reduced JAK-STAT signaling in males downregulated the Amy-p reporter in R4 (Figure 4G), whereas ectopic JAK-STAT signaling was sufficient to induce Amy-p expression in ECs within the clone in the same gut region of females, from which Amy-p is normally absent (Figure 4H). Other gut regions were refractory to JAK-STAT signaling manipulations. We were unable to downregulate endogenous Amy-p in male R2 ECs by downregulating *dome* (Figure 4G) or to ectopically activate it in ECs that do not normally express it outside R4, in either males or females (Figure 4H). Thus, there is a sex-independent restriction in the competence of the midgut to respond to the testis-derived masculinizing signal.

More broadly, we have uncovered inter-organ communication between the male gonad and the gut; the male gonad promotes spatially restricted JAK-STAT signaling in a subset of ECs, leading to male-biased intestinal sugar gene expression in a specific midgut portion.

### Male-Biased Carbohydrate Handling Promotes Food Intake through Secreted Citrate

**Figure S6:**
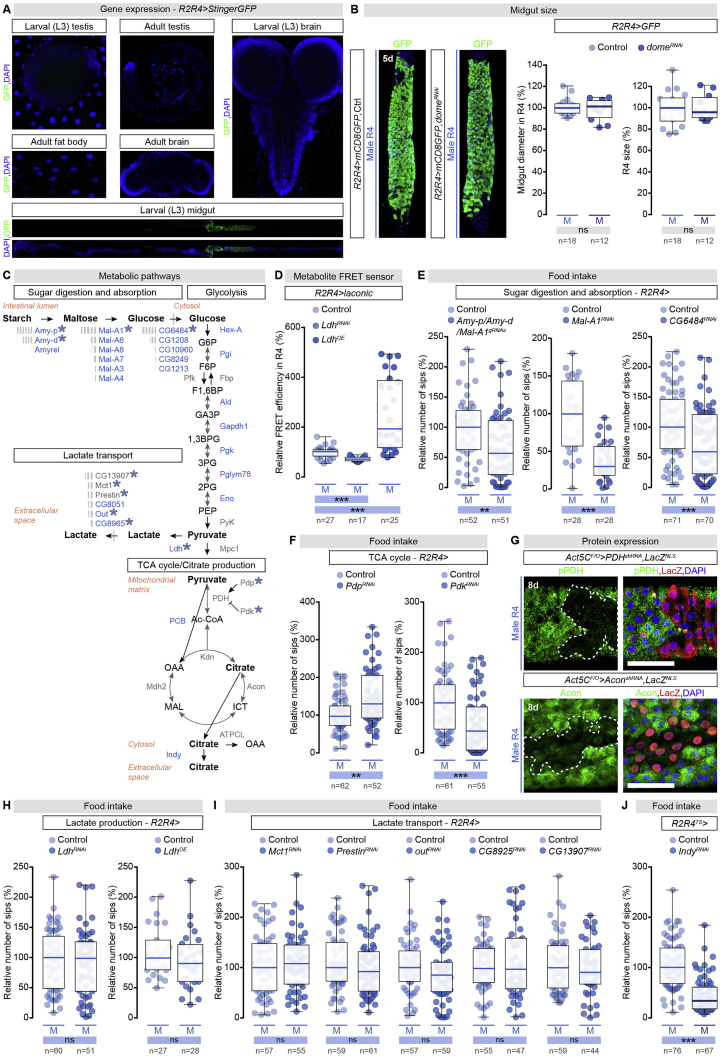
Sex Differences in Intestinal Carbohydrate Handling by Midgut R4 Enterocytes Promote Food Intake through Secreted Citrate, Related to Figure 6. (A) The *R2R4-Gal4* reporter is exclusively expressed in a subset of larval enterocytes. It is absent from testes, brain, and fat body cells (DNA: DAPI, in blue; *R2R4 > StingerGFP*: GFP, in green). (B) Representative images (DNA labeled with DAPI in blue; R4-driven GFP (*R2R4-Gal4 > UAS-mCD8GFP*) is visualized in green) and quantifications of R4 midgut region diameter and size in control males and in males following R2 and R4 enterocyte-specific knockdown of the JAK-STAT receptor *domeless* (*dome*^*RNAi*^). (C) Male-biased enzymes and metabolic pathways in the adult *Drosophila* midgut. Enzymes with male-biased intestinal expression are displayed in blue; enzymes investigated functionally are highlighted with a blue asterisk. Grey bars are proportional to the relative expression levels of each enzyme for enzymes with redundant functions. The specific enzymes from top to bottom are: Amylase proximal (Amy-p), Amylase distal (Amy-d), Maltase A1 (Mal-A1), Maltase A6 (Mal-A6), Maltase A8 (Mal-A8), Maltase A7 (Mal-A7), Maltase A3 (Mal-A3), Maltase A4 (Mal-A4), Hexokinase-A (Hex-A), Phosphoglucose isomerase (Pgi), Phosphofructokinase (Pfk), Fructose-1,6-bisphosphatase (Fbp), Aldolase (Ald), Glyceraldehyde 3 phosphate dehydrogenase 1 (Gapdh1), Phosphoglycerate kinase (Pgk), Phosphoglyceromutase 78 (Pglym78), Enolase (Eno), Pyruvate kinase (PyK), Lactate dehydrogenase (Ldh), Monocarboxylate transporter 1 (Mct1), Outsiders (Out), Mitochondrial pyruvate carrier (Mpc1), Pyruvate dehydrogenase E1 alpha subunit (PDH), Pyruvate dehydrogenase phosphatase (Pdp), Pyruvate dehydrogenase kinase (Pdk), Pyruvate carboxylase (PCB), Knockdown (Kdn), Aconitase (Acon), Malate dehydrogenase 2 (Mdh2), ATP citrate lyase (ATPCL), I’m not dead yet (Indy). The specific metabolites from top to bottom are: glucose-6-phosphate (G6P), fructose-6-phosphate (F6P), fructose-1,6-biphoshate (F1,6BP), glyceraldehyde-3-phosphate (GA3P), 1,3 bisphosphoglycerate (1,3BPG), 3 phosphoglycerate (3PG), 2 phosphoglycerate (2PG), phosphoenolpyruvate (PEP), acetyl coenzyme A (Ac-CoA), isocitrate (ICT), malate (MAL), and oxaloacetate (OAA). (D) Quantification of the FRET signal in R4 enterocytes expressing the laconic lactate sensor from control male midguts, midguts with R2/R4 enterocyte-specific lactate dehydrogenase knockdown (*Ldh*^*RNAi*^), or R2/R4 enterocyte-specific *Ldh* misexpression (*Ldh*^*OE*^). (E) Food intake quantifications based on the number of FlyPAD-monitored sips per male (M) fly following R2 and R4 enterocyte-specific knockdown of the following digestive enzymes and sugar transporter: *Amy-p/Amy-d/Mal-A1*, *Mal-A1* and *CG6484*. For each genetic manipulation in this and all subsequent panels, the median number of sips was arbitrarily set up at 100% for control males, and percentage of that expression is displayed for the other genotypes. (F) Food intake quantifications based on the number of FlyPAD-monitored sips per male (M) fly following R2 and R4 enterocyte-specific knockdown of the *Pdp* and *Pdk* enzymes. (G) Midgut expression of phospho-PDH (pPDH) following clonal knockdown of *PDH* (DNA is labeled in blue with DAPI, anti-beta galactosidase in red is used to stain LacZ-positive cells inside the clone in which PDH knockdown has been induced). Expression of Acon following clonal knockdown of *Acon* (DNA is labeled in blue with DAPI, anti-beta galactosidase in red is used to stain LacZ-positive cells inside the clone in which Acon knockdown has been induced). Expression is reduced within both *PDH* and *Acon* clones, indicative of effective knockdown. (H) Food intake quantifications based on the number of FlyPAD-monitored sips per male (M) fly following R2 and R4 enterocyte-specific knockdown or mis-expression of the *Drosophila* homolog of *Ldh*. (I) Food intake quantifications based on the number of FlyPAD-monitored sips per male (M) fly following R2 and R4 enterocyte-specific knockdown of the following monocarboxylate transporters: *Mct1, Prestin, out, CG8925* and *CG13907*. (J) Food intake quantifications based on the number of FlyPAD-monitored sips per male fly following R2/R4 enterocyte and adult-specific *Indy* knockdown (*R2R4TS > Indy*^*RNAi*^). n denotes the number of flies analyzed for each genotype/condition, except in panels (B) and (D), where n indicates the number of midguts. Scale bars, 50 μm in all images. Asterisks highlighting significant comparisons within male datasets are displayed in blue boxes. See Table S4 for a list of full genotypes.

In mice, the intestine can make glucose *de novo*, which is secreted into the portal vein and can affect hunger and satiety (Soty et al., 2017). We hypothesized that sex differences in intestinal JAK-STAT signaling and sugar handling might similarly affect feeding in flies, perhaps through secretion of a metabolite. To test this idea, we characterized a *Gal4* driver line, *R2R4-Gal4*, expressed exclusively in ECs of the R2 and R4 regions (Figures 4C, 4F, and S6A; see STAR Methods). We used this line to investigate the physiological consequences of abrogating (*Stat92E* downregulation or expression of a dominant-negative *dome*, *UAS-dome*^*ΔCYT*^) or exacerbating (*upd3* overexpression) JAK-STAT signaling in ECs of the midgut R4 region. We also reduced the male bias in JAK-STAT signaling independently from the male gonad in two ways: by depleting the testis from hub cells in *esg*^*shof*^ males (Voog et al., 2014) and by downregulating endogenous *upd1* from the hub by using *fascilin 3 (fas3)-Gal4* (expressed in the hub cells of the testis) (Demarco et al., 2014, Wolfstetter and Holz, 2012). Using flyPAD to monitor feeding behavior in freely behaving flies (Itskov et al., 2014), we observed that reduced JAK-STAT signaling in male ECs resulted in reduced food intake, whereas its upregulation above endogenous levels increased it (Figure 4I). Thus, the JAK-STAT signaling status of male ECs in this sexually dimorphic region controls food intake.

**Figure 5:**
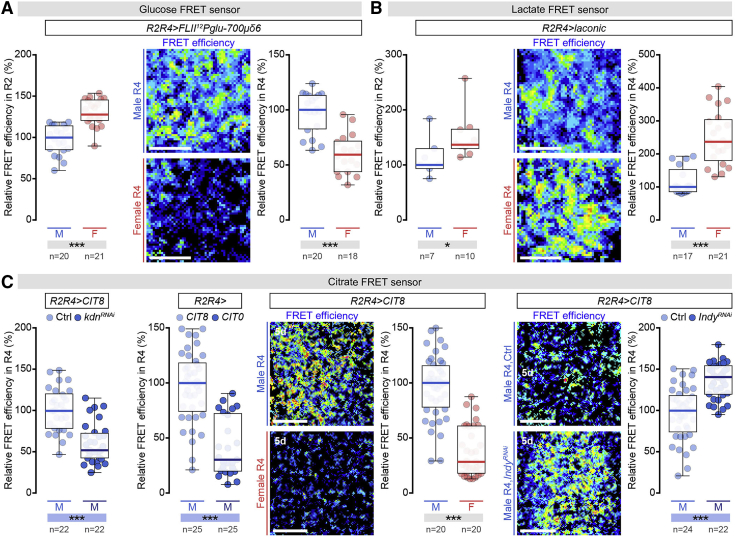
Metabolic Sensors Reveal Sex Differences in Glucose, Lactate, and Citrate Concentrations in R4 Enterocytes (A) Quantification of *FLII*^*12*^*Pglu-700μδ6* glucose sensor FRET signal in R2 (left) and R4 (right) ECs of dissected male (M) and female (F) midguts (quantified on the basis of acceptor photobleaching, see Method Details). In this and subsequent graphs, median FRET ratio for each genotype was arbitrarily set at 100% for control males, and the percentage of that expression is displayed for the other genotypes or sexes. Representative FRET ratio images in R4 shown. (B) Quantification of laconic lactate sensor FRET signal in R2 (left) and R4 (right) ECs of dissected male (M) and female (F) midguts (quantified on the basis of acceptor photobleaching, see Method Details). Representative FRET ratio images in R4 shown. (C) Left: quantification of FRET signal of CIT8 citrate sensor in R4 ECs of control midguts and midguts with R2 and R4 EC-specific KD of citrate synthase *knockdown* (*kdn*^*RNAi*^). Quantification of FRET signal in R4 ECs expressing CIT8 or CIT0 citrate sensors from control male midguts. In the middle is the quantification and representative images of FRET signal in control male and female R4 ECs expressing CIT8 citrate sensor. On the right is the quantification of FRET signal of CIT8 citrate sensor in R4 ECs of control midguts and midguts with R2 and R4 EC-specific KD of plasma membrane citrate transporter *I’m not dead yet (Indy*^*RNAi*^). Representative FRET ratio images in R4 are shown. In all graphs, n = number of midguts analyzed per genotype/condition. Scale bars, 5 μm in all images. Asterisks highlighting significant comparisons across sexes are displayed in gray boxes at bottom of boxplots, those highlighting significant comparisons within male datasets are displayed in blue boxes. See Table S4 for a list of full genotypes.

We hypothesized that male-biased JAK-STAT signaling in ECs would result in the male-biased production and/or secretion of a metabolite. To test this idea, we used genetically encoded FRET-based metabolic sensors expressed specifically in the ECs of R2 and R4, together with a glucose sensor (*UAS-FLII12Pglu-700μδ6*) (Takanaga et al., 2008, Volkenhoff et al., 2018) and a lactate sensor (a *UAS*-based version of the laconic sensor) (San Martín et al., 2013) (see STAR Methods). The glucose sensor revealed higher glucose levels in male than in female ECs of the R4 (but not the R2) region (Figure 5A), consistent with the R4-specific male-biased upregulation of digestive enzymes and sugar transporters.

To monitor lactate levels, we used our validated lactate reporter (Figure S6D) to show that, like glucose, lactate levels were sexually dimorphic in R4 and not in R2. However, lactate levels were lower in male than in female ECs (Figure 5B), suggesting that lactate or an intermediate metabolite “downstream” of glucose was exported out of the EC, or was metabolically diverted.

**Figure 6:**
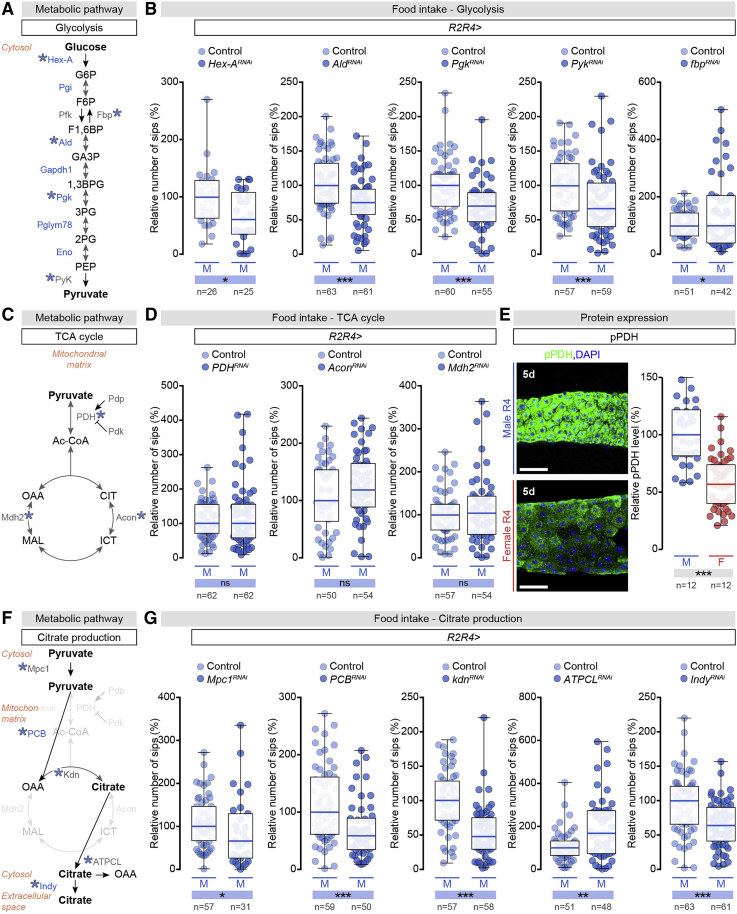
Male-Biased Intestinal Carbohydrate Metabolism Promotes Food Intake through Secreted Citrate (A) Glycolytic pathway enzymes and metabolites. Enzymes in blue have male-biased intestinal expression; enzymes with blue asterisk are tested in (B). Enzymes are abbreviated as follows, top to bottom: Hexokinase-A (Hex-A), Phosphoglucose isomerase (Pgi), Phosphofructokinase (Pfk), Aldolase (Ald), Glyceraldehyde 3 phosphate dehydrogenase 1 (Gapdh1), Phosphoglycerate kinase (Pgk), Phosphoglyceromutase 78 (Pglym78), Enolase (Eno), Pyruvate kinase (PyK), Fructose-1,6-bisphosphatase (Fbp). Metabolites are abbreviated as follows, top to bottom: glucose-6-phosphate (G6P), fructose-6-phosphate (F6P), fructose-1,6-biphoshate (F1,6BP), glyceraldehyde-3-phosphate (GA3P), 1,3 bisphosphoglycerate (1,3BPG), 3 phosphoglycerate (3PG), 2 phosphoglycerate (2PG), phosphoenolpyruvate (PEP). (B) Food intake quantifications based on FlyPAD-monitored sips per male fly after R2 and R4 EC-specific glycolytic enzyme KD: *Hex-A*, *Ald*, *Pgk*, *PyK*, and *fbp*. For all graphs, median number of sips was arbitrarily set at 100% for control males and the percentage of that expression is displayed for other genotypes. (C) Tricarboxylic acid (TCA) cycle enzymes and metabolites. Enzymes with blue asterisk tested in (D). Enzymes are abbreviated as follows, top to bottom: Pyruvate dehydrogenase E1 alpha subunit (PDH), Pyruvate dehydrogenase phosphatase (Pdp), Pyruvate dehydrogenase kinase (Pdk), Aconitase (Acon), Malate dehydrogenase 2 (Mdh2). Metabolites, top to bottom: acetyl coenzyme A (Ac-CoA), citrate (CIT), isocitrate (ICT), malate (MAL), oxaloacetate (OAA). (D) Food intake quantifications based on FlyPAD-monitored sips per male fly after R2 and R4 EC-specific KD of TCA cycle enzymes: *PDH*, *Acon*, *Mdh2*. (E) Quantification of expression level of phospho-PDH (pPDH) protein in R4 region of adult male (M) and female (F) midguts. Representative images are shown (nuclei: blue, DAPI; pPDH, green). (F) Enzymes and metabolites of the pyruvate/citrate cycle. Enzymes in blue have male-biased intestinal expression; enzymes with blue asterisk are tested in (G). Enzymes are abbreviated as follows, top to bottom: Mitochondrial pyruvate carrier (Mpc1), Pyruvate carboxylase (PCB), Knockdown (Kdn), ATP citrate lyase (ATPCL), *Drosophila* plasma membrane citrate efflux transporter I’m not dead yet (Indy). (G) Food intake quantifications based on FlyPAD-monitored sips per male fly after R2 and R4 EC-specific KD of: *Mpc1*, *PCB*, *kdn*, *ATPCL, Indy*. n = fly numbers analyzed per genotype, except in (E), where n = midguts. Scale bars, 50 μm in all images. Asterisks highlighting significant comparisons across sexes are displayed in gray boxes at bottom of graphs, whereas those highlighting significant comparisons within male datasets are displayed in blue boxes. See Table S4 for a list of full genotypes. See also Figures S6 and S7.

To test this idea, we used food intake as a behavioral readout for a genetic screen in which we knocked out male-biased intestinal sugar genes, reasoning that KD of any enzymes mediating conversions “upstream” of this metabolite or those involved in its transport out of the EC would reduce food intake, whereas KD of “downstream” enzymes would have no effect (or increase food intake if their normal function was to divert the use of this metabolite to other intracellular pathways). R2- and R4-specific KD of genes for enzymes involved in sugar digestion, absorption, and glycolysis (alone or in combination, see STAR Methods) all reduced food intake (Figures 6A, 6B, S6C, and S6E), suggesting that the key metabolite was the glycolytic end-product pyruvate or a downstream metabolite.

Interference with the enzymes mediating pyruvate to lactate conversion, its subsequent transport, or the pyruvate dehydrogenase complex mediating its decarboxylation into acetyl coenzymeA (acetyl-CoA) for mitochondrial oxidation (see STAR Methods), all failed to affect food intake (Figures 6C, 6D, S6C, S6H, and S6I), arguing against anaerobic glycolysis and the oxidative entry into the tricarboxylic acid (TCA) cycle being the source of the male-biased production and/or secretion of a metabolite. Consistent with this idea, immunostaining analysis revealed higher levels of phosphorylated Pyruvate dehydrogenase E1 alpha subunit (PDH) (i.e., inactive) (Seegmiller et al., 2002, Korotchkina and Patel, 2001, Linn et al., 1969) in the R4 region of male flies than in female flies (Figure 6E), and KD of genes coding for TCA cycle enzymes did not affect food intake (Figures 6C, 6D, and S6G).

A third way in which pyruvate is utilized involves the anaplerotic pyruvate carboxylase (PCB)-mediated pathway leading to citrate production through the pyruvate/citrate cycle (Iacobazzi and Infantino, 2014, Jensen et al., 2008), and involves PCB-mediated production of oxaloacetate (OAA), which is then converted to citrate by citrate synthase (Knockdown (Kdn) in *Drosophila*) (Fergestad et al., 2006). Genetic manipulations predicted to interfere with this route of citrate production reduced food intake. These included *Mitochondrial pyruvate carrier 1* (*Mpc1*) (Bricker et al., 2012) KD, expected to reduce pyruvate import into the mitochondria, and *kdn* or *PCB* (Camporeale et al., 2007) KD, reducing its subsequent conversions (Figures 6F and 6G). Similarly, modulating the amount of pyruvate available for citrate production by forcing or inhibiting its conversion to Acetyl-CoA also affected food intake in both directions. KD of the PDH inhibitory kinase (Pyruvate dehydrogenase kinase, Pdk) (Katsube et al., 1997), which was predicted to increase acetyl-CoA production and thereby reduce pyruvate available for citrate production, reduced food intake (Figures S6C and S6F). In contrast, KD of the PDH-activating phosphatase (Pyruvate dehydrogenase phosphatase, Pdp) (Chen et al., 2006), which was predicted to have the opposite effects on acetyl-CoA production and pyruvate availability, increased food intake (Figures S6C and S6F). These findings suggest that citrate is the key secreted metabolite downstream of JAK-STAT signaling in mediating systemic effects on food intake.

We tested this further by downregulating *ATP citrate lyase* (*ATCPL*) (Ryerse et al., 1997), which converts citrate to OAA. We predicted that this would increase citrate levels available for export, and consistent with this idea, we observed increased food intake (Figures 6F and 6G). Conversely, downregulation of the *I’m not dead yet* (*Indy*) transporter (Rogina et al., 2000), known to transport citrate (Inoue et al., 2002a, Knauf et al., 2002), reduced food intake (Figures 6F and 6G). Adult-confined *Indy* KD further confirmed a role for citrate in promoting food intake in adult males (Figure S6J). To confirm that citrate is the key secreted metabolite downstream of JAK-STAT signaling, we generated a genetically encoded nanosensor for real-time *in vivo* quantification of citrate levels (CIT8) (Ewald et al., 2011) (see Method Details) and validated its function and specificity (Ewald et al., 2011) (Figure 5C; see Method Details). Using the sensor, we found that citrate levels were sexually dimorphic in R4; male ECs have 2.5 times more citrate than female ECs (Figure 5C). Monitoring citrate levels following *Indy* KD revealed increased citrate levels in male R4 ECs (Figure 5C), confirming that *Indy* normally transports citrate out of these cells.

**Figure S7:**
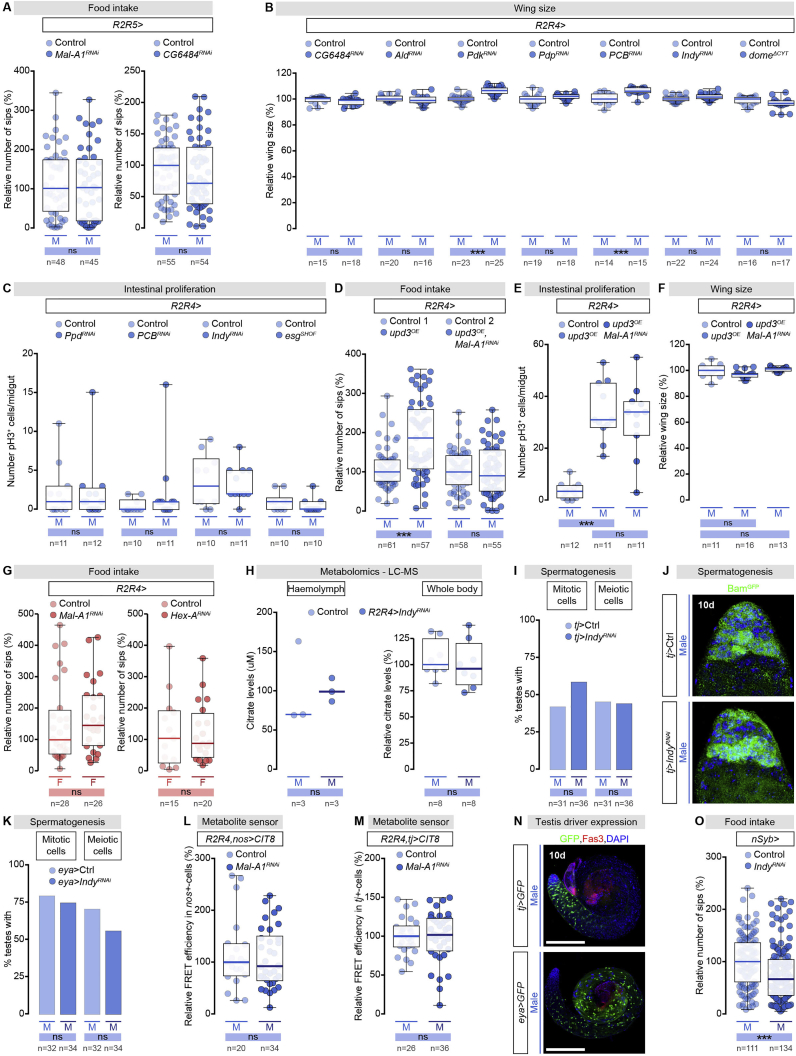
Male-Biased Carbohydrate Metabolism Is Genetically Downstream of the JAK-SAT Signaling in Enterocytes of the R4 Region and can be Uncoupled from Larval Growth and Intestinal Proliferation, Related to Figures 5 and 6 and 7 (A) Food intake quantifications based on the number of FlyPAD-monitored sips per male (M) fly following R2 and R5 enterocyte-specific knockdown of the *Maltase-A1* (*Mal-A1*) and *CG6484* enzymes. Downregulation of intestinal sugar genes sugar genes in R2 and R5 does not affect male food intake. (B) Adult wing size quantifications (used as a measurement of body size, (Shingleton et al., 2009, Shingleton et al., 2017)) for male (M) flies following enterocyte-specific knockdown of the following enzymes: *CG6484*, *Aldolase* (*Ald*), *Pyruvate dehydrogenase kinase* (*Pdk*), *Pyruvate dehydrogenase phosphatase* (*Pdp*), *Pyruvate carboxylase* (*PCB*), *I’m not dead yet* (*Indy*) and *domeless*^*ΔCYT*^ (*dome*^*ΔCYT*^). Downregulation of intestinal sugar genes sugar genes or JAK-STAT signaling in R2 and R4 does not reduced male body size. (C) Intestinal proliferation quantified as the number of pH3-positive cells in male midguts following enterocyte-specific knockdown of the following enzymes: *Pdp*, *PCB*, *IIndy* and for the *shutoff* (*esg*^*SHOF*^) mutation. Downregulation of intestinal sugar genes sugar genes in R2 and R4 does not impact male intestinal proliferation. (D) Food intake quantifications based on the number of FlyPAD-monitored sips per male (M) fly following R2 and R4 enterocyte-specific mis-expression of the JAK-STAT ligand *unpaired 3 (udp3)* alone or in combination with *Mal-A1* downregulation. The increased food intake resulting from *upd3* overexpression in male enterocytes can be reduced to wild-type levels by simultaneous downregulation of *Mal-A1*. (E) Intestinal proliferation quantified as the number of pH3-positive cells in male midguts following enterocyte-specific mis-expression of the JAK-STAT ligand *udp3* alone or in combination with *Mal-A1* downregulation. In contrast to its effect on food intake, *Mal-A1* downregulation fails to reduce the increased stem cell proliferation observed following *upd3* overexpression. (F) Body size assessments based on adult wing size quantifications for male flies following R2 and R4 enterocyte-specific mis-expression of the JAK-STAT ligand *udp3* alone or in combination with *Mal-A1* downregulation. Concurrent over-activation of JAK-STAT signaling in ECs (by ectopic *upd3* expression) and downregulation of the intestinal sugar gene *Mal-A1* reduces food intake without affecting body size. (G) Food intake quantifications based on the number of FlyPAD-monitored sips per female (F) fly following R2 and R4 enterocyte-specific knockdown of the *Mal-A1* and *Hexokinase-A (Hex-A)* enzymes. Downregulation of intestinal sugar genes does not affect female food intake. (H) LC-MS quantifications of hemolymph (left graph) and whole-body (right graph) citrate in control males and in males following R2/R4 enterocyte-specific knockdown of the plasma membrane Indy citrate transporter. Intestinal *Indy* knockdown has no impact on circulating or whole body citrate. (I) Quantifications of the number of mitotic and meiotic pH3-positive germ cells in control testes and in testes following testis-specific *Indy* knockdown from the *traffic jam (tj)-Gal4* driver line. (J) Expression pattern of Bag of marbles (Bam) (visualized in green using the Bam^GFP^ protein reporter) in control testes and in testes following testis-specific *Indy* knockdown with the *tj-Gal4* reporter line. Representative images are shown (DNA is labeled with DAPI in blue). (K) Quantifications of the number of mitotic and meiotic pH3-positive germ cells in control testes and in testes following testis-specific *Indy* knockdown from the *eyes absent (eya)-Gal4* driver line. (L, M) Quantification of the CIT8 citrate sensor’s FRET signal in germline stem cells (*nanos (nos)-Gal4*-positive) (L) or early-stage somatic cells (M) of testes of control males or males with R2/R4 enterocyte-specific knockdown of *Maltase-A1* (*Mal-A1*^*RNAi*^). (N) The *tj-Gal4* and *eya-Gal4* reporters are selectively expressed in early-stage and late-stage somatic cells of the testis (respectively), and not in the germ cells (DNA: DAPI, in blue; *tj/eya > StingerGFP*: GFP, in green; hub cells: Fasciclin 3 (Fas3), in red). (O) Food intake quantifications based on the number of FlyPAD-monitored sips per male fly following neuronal-specific *Indy* downregulation (*neuronal Synaptobrevin (nSyb) > Indy*^*RNAi*^). n denotes the number of flies (A, D, G and H), wings (B and F), midguts (C and E) or testes (I, K, L, M and O). Scale bars, 200 μm in all images. Asterisks highlighting significant comparisons within female and male datasets are displayed in red and blue boxes respectively. See Table S4 for a list of full genotypes. See also Table S2.

Finally, we conducted a series of additional controls to validate our findings. We showed that the R2 region does not contributte to these phenotypes (Figure S7A) and that possible developmental effects of downregulating intestinal JAK-STAT signaling or the sugar genes on body or gut size did not underlie the differences in food intake (Figures S6B and S7B). We also ruled out that the food intake phenotypes resulted from effects of JAK-STAT signaling or intestinal sugar gene expression on intestinal stem cell proliferation. Indeed, most manipulations that abrogated the male bias in intestinal sugar gene expression and reduced food intake (e.g., testis hub loss or EC-specific *Indy* knockdown) did not affect male stem cell proliferation (Figure S7C). In the few instances where stem cell proliferation was increased (following over-activation of JAK-STAT signaling in ECs by ectopic Upd3 expression) (Osman et al., 2012), the proliferation increase could be uncoupled from the effect on food intake by simultaneously downregulation of an intestinal sugar gene (*Mal-A1*), which reduced food intake without reducing stem cell proliferation to basal levels (Figures S7D, S7E, and S7F). This experiment provides further support for the model that male-biased carbohydrate metabolism is genetically “downstream” of the male bias in JAK-SAT signaling in ECs of the R4 region. Finally, reducing citrate production in R2 and R4 in females (by downregulating *Mal-A1* or *Hex-A* enzymes) had no effect on their feeding behavior (Figure S7G) indicating that modulation of feeding by the pyruvate/citrate cycle activity in ECs is male-specific.

Together, our data support a model whereby male-biased activation of JAK-STAT signaling in ECs of the R4 region upregulates intestinal sugar gene expression to produce cytosolic citrate, which is exported into the circulation by the citrate transporter Indy to promote food intake.

### Intestinal Citrate Efflux Is Required for Testis Germline Maturation

**Figure 7:**
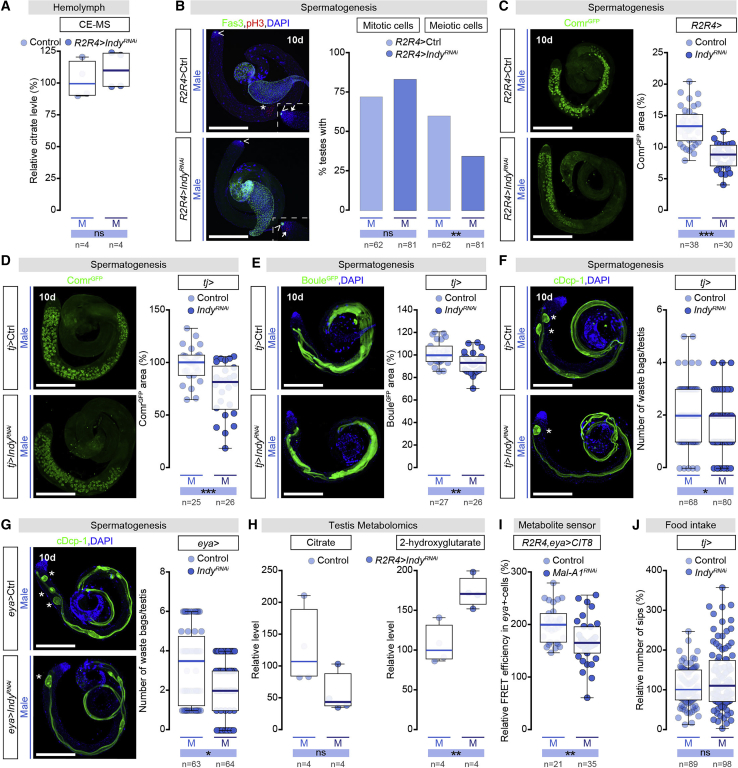
Intestinal Citrate Efflux Is Required for Testis Germline Maturation (A) CE-MS measurement of hemolymph citrate in control males and in males after R2 and R4 EC-specific KD of plasma membrane Indy citrate transporter. n = 4 samples, each containing hemolymph from 120–280 flies. (B) Immunohistochemical analysis of testis anatomy and germline maturation based on expression of Fasciclin 3 (Fas3, labeling hub cells (white arrowhead), green), DAPI (blue) and number of phospho Histone3 (pH3)-positive cells (staining mitotic (arrow) and meiotic (asterisk) cells, red) in testes of control males and males with R2 and R4 EC-specific *Indy* KD. (C) Quantification of Cookie monster (Comr) expression pattern (Comr^GFP^, green) in control testes and testes after R2 and R4 EC-specific *Indy* KD. Representative images shown (DNA: DAPI, blue). (D–F) Representative images (DNA: DAPI, blue; protein, green) and quantifications of Comr^GFP^ (D), Boule^GFP^ (E), cleaved Dead caspase-1 (Dcp-1) (F) expression in testes of control males and in males after testis-specific *Indy* KD (*traffic jam (tj)-Gal4* line). (G) Quantification of cleaved Dcp-1 expression (green) in control testes and testes after testis-specific *Indy* KD (*eyes absent (eya)-Gal4* line). Representative images shown (DNA: DAPI, blue). (H) GC-MS measurements of the testis concentration of citrate and 2-hydroxyglutarate (2HG) in control males and in males after R2 and R4 EC-specific *Indy* KD. n = 4 samples, each containing 150 dissected testes. (I) Quantification of FRET signal in late somatic testis cells expressing CIT8 from control males or males with R2 and R4 EC-specific KD of *Maltase-A1* (*Mal-A1*^*RNAi*^). (J) Food intake quantification based on FlyPAD-monitored sips per male fly after testis-specific *Indy* KD (*tj-Gal4* line). n = testes number analyzed per genotype except for in (J), where n = fly number analyzed. Scale bars, 200 μm in all images. Asterisks highlighting significant comparisons across male datasets are displayed in a blue box. See Table S2 for metabolites/concentrations. See Table S4 for a list of full genotypes. See also Figure S7 and Table S2.

To investigate possible roles of male-specific intestinal citrate efflux, we quantified citrate levels in both hemolymph and whole flies, via liquid chromatography-mass spectrometry (LC-MS) and capillary electrophoresis mass spectrometry (CE-MS) (see Method Details), and observed high levels of circulating citrate in male flies (100.5 ± 54.3 μM) (Figure S7H), but neither this circulating citrate nor whole-body citrate levels were significantly reduced by preventing intestinal citrate efflux (by R2R4-driven *Indy* KD) (Figures 7A and S7H; Table S2). CE-MS analysis of hemolymph revealed no large-scale effects on other circulating metabolites after intestinal *Indy* KD (Table S2). We hypothesized that the testis might utilize gut-derived citrate. To test this idea, we downregulated the *Indy* citrate efflux transporter specifically in R2 and R4 intestinal ECs via *R2R4-Gal4* and assessed the consequences in the testis. Immunohistochemical analysis indicated that downregulation of intestinal citrate efflux had little effect on testis tissue architecture and DNA replication (assayed with phospho-Histone 3 [pH3]) (Tan et al., 2017, Tapia et al., 2006) (Figure 7B). However, pH3 quantification revealed that, although there were no obvious differences in mitoses in the tip region where spermatogonia are generated from stem cells (Greenspan et al., 2015), pH3 numbers were substantially reduced in the region in which spermatids are produced from spermatogonia, consistent with a delay in gamete maturation (Figure 7B). This was confirmed by Cookie monster (Comr^GFP^) labeling of primary spermatocyte nuclei (Jiang and White-Cooper, 2003), which revealed a reduction in spermatocyte number after intestinal *Indy* KD (Figure 7C).

We hypothesized that impaired intestinal citrate efflux might contribute to delayed gamete maturation through metabolic changes in the testis. To explore this idea, we reduced citrate import in testes by testis-specific *Indy* KD in testes early-stage somatic cells (Figure S7N) and saw no effect on mitotic spermatogonia (Figures S7I and S7J) but reduced numbers of primary spermatocytes (Figure 7D), elongating spermatids (Figure 7E), and individualizing spermatids (Figure 7F), mirroring the phenotype obtained by reducing intestinal citrate efflux. Confining *Indy* KD to late-stage somatic cells (Figure S7N) also reduced the numbers of individualizing spermatids (Figure 7G) without affecting mitotic spermatogonia (Figure S7K). These genetic experiments also uncoupled the roles of gut-derived citrate in sustaining sperm production from its role in stimulating appetite; reducing testis citrate import (by means of *traffic jam* (*tj)*-driven *Indy* KD) impaired spermatogenesis without affecting food intake (Figures 7D–7F and 7J). Reduced food intake was, conversely, apparent when *Indy* was selectively downregulated in neurons (Figure S7O).

To more directly test whether gut-to-testis citrate transfer sustains spermatogenesis, we analyzed adult testes via gas chromatography mass spectrometry (GC-MS), comparing adult testes from control male flies to those from male flies in which the Indy citrate efflux transporter had been specifically downregulated in intestinal ECs of the R2 and R4 regions. We observed a trend toward reduced citrate levels in testes samples after intestinal knockdown, consistent with reduced exogenous supply of citrate to the testis (Figure 7H; Table S2). Impaired intestinal citrate efflux also resulted in a significant accumulation of 2-hydroxyglutarate (2HG) in testes (Figure 7H; Table S2). 2HG is an oncometabolite (Chowdhury et al., 2011, Figueroa et al., 2010, Losman and Kaelin, 2013, Lu et al., 2012, Xu et al., 2011), but is also produced by healthy tissues, where it can accumulate when cytosolic citrate is low (Li et al., 2017, Li et al., 2018, Nota et al., 2013, Palmieri, 2013, Ye et al., 2018).

We monitored citrate levels in different testis cell types by expressing our citrate sensor in both gut and testis cells, while simultaneously preventing male-specific intestinal citrate production in R2 and R4 ECs via *Mal-A1*^*RNAi*^ (we chose *Mal-A1* because its expression is highly specific to the midgut and entirely absent from testes) (Leader et al., 2018) (and data not shown). Reduced gut-derived citrate production resulted in a significant reduction in citrate intracellular levels selectively in testis late-stage somatic cells (Figure 7I) but not in germline stem cells (Figure S7L) or early-stage somatic cells (Figure S7M) (for testis cell-type-specific reporter expression, see Figures 3B and S7N). Together, these results indicate that intestinal citrate is locally transferred via the Indy transporter from the R4 midgut region to the adjacent testis, where it sustains maturation of male gametes.

## Discussion

### Sex Differences in Intestinal Carbohydrate Metabolism

Regional differences in gene expression are observed along animal gastrointestinal tracts, suggestive of functional specializations (Bates et al., 2002, Haber et al., 2017). We now provide evidence for region- and cell-type-specific carbohydrate metabolism. Intestinal carbohydrate metabolism also differs between the sexes, illustrating how sex differences can be confined to specific organ portions; even when digestive enzymes are more broadly expressed along the midgut, their male upregulation is posterior midgut (R4)-specific. We suggest that specific gut portions might be physiologically “sexualized” to subserve reproductive needs—in this case spermatogenesis. The posterior midgut might be more broadly sexually dimorphic than other intestinal regions; oxidative stress response proteins are male biased and Yp1 is female biased in this same region (Hudry et al., 2016) (Figure 1). In female flies, posterior midgut ECs adjust their lipid metabolism after mating to maximize reproductive output (Reiff et al., 2015). It will be of interest to explore whether this requires their female identity; if it does, is female identity the “ground state” in the absence of a male gonad, or does it result from an ovary signal? Comparative studies could also explore contributions of intestinal sex differences to reproductive success in animals other than *Drosophila* and whether the evolution of a placenta (an organ purpose-built for reproduction) replaced or reinforced such intestinal contributions in female mammals.

### Gonadal Control of Intestinal Sexual Identity

The male gonad controls sex differences in intestinal carbohydrate metabolism through male-biased cytokine signaling activity. *Drosophila* Upd belong to the type I family of cytokines, like mammalian interleukins and leptin. In both humans and rodents, leptin expression is sexually dimorphic (Couillard et al., 1997, Gui et al., 2004, Havel et al., 1996, Landt et al., 1998, Montague et al., 1997, Rosenbaum et al., 1996, Saad et al., 1997). Males and females also differ in their interleukin repertoire, which contributes to sex differences in immunity and autoimmune disease (Russi et al., 2018, Voigt et al., 2016, Xiong et al., 2015). A possible contribution of cytokines such as leptin to sex differences in organ physiology deserves further investigation, particularly in light of leptin’s known reproductive and gastrointestinal roles (Sáinz et al., 2015, Smith et al., 2002).

The gonadal regulation of intestinal sugar metabolism contrasts with the intrinsic, sex-chromosome-dependent control of sex differences in gut stem cell proliferation (Hudry et al., 2016). This illustrates the complexity of an organ’s “sexual identity;” two lineage-related cells within an epithelium (stem cells and their EC progeny) acquire sex-specific functions (proliferation and carbohydrate metabolism) through two distinct mechanisms. Sexual identity is reversible in both cases and needs to be actively maintained in adults, raising the question of whether adult plasticity in sexual identity might be adaptive. Environmental factors could modulate the expression or penetrance of sex determinants—possibly tissue specifically. There is some evidence in support of this idea—male flies that lack Fru^M^ are defective in courtship but learn to court when housed in groups with wild-type flies in a Dsx^M^-dependent manner (Pan and Baker, 2014). Early life exposure to nutrient scarcity also affects neuronal wiring selectively of male *C. elegans* (Bayer and Hobert, 2018). In light of these and our findings, it will be of interest to explore how plastic sex differences in physiology are and why.

### Inter-organ Metabolic Communication

Gut-gonad communication is bi-directional; the male gonad communicates with a specific gut portion, which responds by secreting citrate. Gut-derived citrate in turn promotes food intake and maturation of male gametes. How might it do so? Import of exogenous citrate might help sustain the high TCA cycle requirements of developing sperm (Bajpai et al., 1998, Boussouar and Benahmed, 2004). Sertoli cells are highly glycolytic and have been proposed to act as a paracrine source of lactate for developing gametes (Boussouar and Benahmed, 2004, Oliveira et al., 2015). It is therefore conceivable that citrate acts as another exogenous carbon source. Consistent with this idea, the mitochondrial citrate carrier is present and active in human sperm (Cappello et al., 2012), and boar sperm can metabolize exogenous citrate through the Krebs cycle *in vitro* (Medrano et al., 2006). Alternatively, import of gut-derived citrate might sustain membrane formation through its conversion to acetyl-CoA by ATCPL, then used for fatty acid synthesis; both spermatid elongation and individualization require extensive membrane biosynthesis and remodeling (Laurinyecz et al., 2016, Szafer-Glusman et al., 2008). Citrate could also support epigenetic changes relevant to male gamete maturation through its conversion to acetyl-CoA, used as a donor for histone acetyl transferase-mediated histone acetylation (Su et al., 2016).

The effects of gut-derived citrate on sperm production can be uncoupled from its orexigenic actions. Preventing citrate import into neurons reduces food intake, suggesting that its promotion of feeding might result from its actions in the nervous system. Given that preventing gut-derived citrate efflux does not affect circulating citrate, it is tempting to speculate that local gut and/or testis-innervating neurons might harbor the citrate sensors. This effect of citrate on food intake is male specific—reducing gut-derived citrate efflux does not reduce feeding in females. Our ongoing work is revealing that, in females, gonad to gut communication also promotes feeding, but via a different mechanism and possibly as a result of different dynamics and/or metabolic requirements of male and female gamete production (D. Hadjieconomou, unpublished data).

More generally, our study provides evidence that citrate functions in communication between organs. In mammals, plasma levels of citrate are among the highest among TCA cycle intermediates (Costello and Franklin, 1991a, Costello and Franklin, 2016b, Hui et al., 2017, Mycielska et al., 2009). Organ-specific differences in citrate production and consumption have been reported (Jang et al., 2019), but little is known about its roles and regulation by diet, age, or sex. Bone—an organ that controls male fertility through an endocrine hormone— produces unusually high amounts of citrate (Costello et al., 2012, Dickens, 1941, Oury et al., 2011). In the context of male gametes, the prostate should also be considered as a potentially relevant citrate source; it secretes large amounts of citrate into the seminal fluid that developing sperm will come into contact with (Costello and Franklin, 1991a, Mycielska et al., 2009). The roles of prostate citrate have been investigated in the context of the metabolic rewiring of prostate tumors (Costello and Franklin, 1991b, Costello and Franklin, 2016a). Less is known about its roles in the context of sperm production, partly because surgical interventions such as prostatectomy impair other aspects of testis physiology. Contributions of exogenous citrate to sperm-mediated transgenerational effects also deserve further investigation in light of citrate’s epigenetic effects. It will also be of interest to characterize the transporters for citrate import into the germline to control spermatogenesis and/or into neurons to control food intake; *CG7309* and *Indy-2* genes code for putative citrate transporters and have testis-specific expression (Leader et al., 2018). In mammals, the Indy homolog NaCT is specifically expressed in testis, liver, and brain (Inoue et al., 2002b), and NaCT knockout mice are protected from diet- and age-induced adiposity and insulin resistance (Birkenfeld et al., 2011).

The physical proximity between the male gonad and the gut portion to which it signals raises the possibility that the relative positioning of internal organs is physiologically significant. Although this particular association is not conserved in adult humans, testis development is a complex process from a three-dimensional perspective, which in all placental mammals involves descent of testes from a position near the kidneys (Sharma et al., 2018), perhaps providing opportunities for inter-organ communication. More generally, a spectrum of conditions (so-called heterotaxy syndromes) resulting from the abnormal arrangement of internal organs including the gastrointestinal tract can lead to serious disease manifestations. Subtler, likely undiagnosed defects in intestinal positioning could result in milder gastrointestinal symptoms and/or contribute to differences in whole-body physiology across individuals.

## STAR★Methods

### Key Resources Table

**Table:** 

REAGENT or RESOURCE	SOURCE	IDENTIFIER
**Antibodies**		

Chicken anti-GFP, 1/10000	Abcam	Cat#ab13970; RRID: AB_300798
Mouse anti-GFP, 1/1000	Roche	Cat#11814460001; RRID: AB_390913
Chicken anti-beta Galactosidase, 1/200	Abcam	Cat#ab9361; RRID: AB_307210
Rabbit anti-phospho-Histone H3 Ser10, 1/500	Cell Signaling Technology	Cat#9701L; RRID: AB_331535
Mouse anti-Fas3, 1/50	DSHB	Cat#7G10; RRID: AB_528238
Rabbit anti-Pyruvate dehydrogenase E1-alpha subunit (phospho S293), 1/200	Abcam	Cat#ab92696; RRID: AB_10711672
Rabbit anti-Aconitase 2, 1/200	Abcam	Cat#ab83528; RRID: AB_1859827
Rabbit anti-cleaved *Drosophila* Dcp-1 (Asp216), 1/500	Ozyme	Cat#9578S; RRID: AB_2721060
Rhodamine Phalloidin, 1/1000	ThermoFisher scientific	Cat#R415; RRID: AB_2572408

**Deposited Data**		

Raw RNaseq data	Hudry et al., 2016	GEO: GSE74775

**Experimental Models: Organisms/Strains**		

*D. melanogaster* lines – See Table S4	Various	N/A

**Oligonucleotides**		

RT-qPCR primers – See Table S3	This paper	N/A

**Software and Algorithms**		

Fiji	PMID: 22743772	https://fiji.sc/
Adobe Illustrator CC 2018	Adobe.com	N/A
Prism 7 GraphPad	GraphPad Software	https://www.graphpad.com/scientific-software/prism/

### Lead Contact and Materials Availability

Further information and requests for resources and reagents (such as newly generated *Drosophila* stocks) should be directed to and will be fulfilled by the Lead Contact, Irene Miguel-Aliaga (i.miguel-aliaga@imperial.ac.uk).

### Experimental Models and Subject Details

#### Fly husbandry

Fly stocks were reared on a standard cornmeal/agar diet (6.65% cornmeal, 7.15% dextrose, 5% yeast, 0.66% agar supplemented with 2.2% nipagin and 3.4 mL/L propionic acid). All experimental flies were kept in incubators at 25°C, 65% humidity and on a 12 hr light/dark cycle. Flies were transferred to fresh vials every 3 days, and fly density was kept to a maximum of 15 flies per vial. 5-day old virgin flies were used unless otherwise indicated.

For metabolomics and testes immunostainings, males were aged for 10 days before dissection. For clonal analyses (flip-out clones), 3-day-old adults (raised and aged at 25°C) were heat-shocked for 12 min at 37°C to induce clones, and were then kept at 25°C for 5 days until dissection. Flies were transferred to fresh vials every 3 days.

#### Fly stocks

##### Reporters

Mal-A1^GFP^ (VDRC: 318296), Mal-A3^GFP^ (this study), Treh^GFP^ (BDSC: 59825), Hex-A^GFP^ (VDRC: 318587), Pgi^GFP^ (this study), Amy-p^GFP^ (this study), Ldh^GFP^ (gift from U. Banerjee, YD0852, generated by Quiñones-Coello et al., 2007), *Stat92E-GFP* (BDSC: 26199), *GstD1-GFP* (gift from U. Banerjee, generated by Sykiotis and Bohmann, 2008), Yp1^GFP^ (VDRC: 318746), Comr^GFP^ (VDRC: 318559), Boule^GFP^ (BDSC: 64431), bam^GFP^ (VDRC: 318001).

##### *Gal4* drivers

*R2R4-Gal4* (this study, enhancer VT004416: a 2541 base pair fragment from the flanking non-coding or intronic region of *LManVI* fused upstream of a *Drosophila* synthetic core promoter (DSCP) followed by a sequence encoding a *Gal4* driver, (Kvon et al., 2014)), *esg-Gal4*^*NP7397*^ (gift from J. de Navascués), *mex1-Gal4* (Phillips and Thomas, 2006), *Mal-A7-Gal4* (this study, see below for details), *nos-Gal4* (BDSC: 4937), *Act5C-Gal4* (BDSC: 4414), *tj-Gal4* (DGGR: 104055), *Act5C-FRT-y-FRT-GAL4* (BDSC: 4410), *Fas3-Gal4* (DGGR: 103948), *da-Gal4* (BDSC: 55851), *pros*^*V1*^*-Gal4* (Balakireva et al., 1998), *Myo1A-Gal4* (DGGR: 112001), *vm-Gal4* (BDSC: 48547), *elav-Gal4* (BDSC: 458), *repo-Gal4* (BDSC: 7415), *Lpp-Gal4* (Brankatschk and Eaton, 2010), *Hml-Gal4* (BDSC: 30139), *Akh-Gal4* (BDSC: 25683), *Aug21-Gal4* (BDSC: 30137), *eya-Gal4* (*eyaA3-GAL4*, gift from M. Amoyel, generated by Leatherman and Dinardo, 2008), *tubP-Gal80*^*TS*^ (BDSC: 7019), *R2R5-Gal4* (DGGR: 112920, generated by Buchon et al., 2013).

##### *UAS* transgenes

*UAS-StingerGFP* (BDSC: 65402), *UAS-mCD8GFP* (*UAS-IVS-mCD8GFP*, BDSC: 32186), *UAS-FLII12Pglu-700μδ6* (Volkenhoff et al., 2018), *UAS-laconic* (this study, see below for details), *UAS-Ldh* (FlyORF: F002924), *UAS-flp* (BDSC: 4539), *UAS-tra*^*F*^ (BDSC: 4590), *UAS-upd3-GFP* (Wang et al., 2014), *UAS-hop*^*Tum*^ (gift from E. Bach, generated by Harrison et al., 1995), *UAS-dCas9*^*VPR*^ (BDSC: 67052), *UAS-dome*^*ΔCYT*^ (Brown et al., 2001), *UAS-dcr2* (VDRC: 60010), *UAS-InR*^*DN*^ (BDSC: 8252), *UAS-gRNAS Amy-p/Amy-d/Mal-A1* (this study, see below for details), *UAS-Cas9* (BDSC: 54594), *UAS-Ldh*^*RNAi*^ (BDSC: 33640), *UAS-tra*^*RNAi*^ (BDSC: 28512), *UAS-snf*^*RNAi*^ (BDSC: 55914), *UAS-Sxl*^*RNAi*^ (BDSC: 38195), *UAS-GFP* (BDSC: 35786), *attP2* control line (BDSC: 36303), *attP40* control line (36304), *GD* control line (VDRC: 60000), *KK* control line (VDRC: 60100), *UAS-dome*^*RNAi*^ (BDSC: 34618), *UAS-hop*^*RNAi*^ (VDRC: GD 40037), *UAS-Stat92E*^*RNAi*^ (BDSC: 31318), *UAS-upd1*^*RNAi*^ (BDSC: 28722), *UAS-upd1*^*OE*^ (BDSC: 67555), *UAS-Hex-A*^*RNAi*^ (VDRC: KK 104680), *UAS-Ald*^*RNAi*^ (BDSC: 65884), *UAS-Pgk*^*RNAi*^ (VDRC: KK 110081), *UAS-PyK*^*RNAi*^ (VDRC: GD 49533), *UAS-fbp*^*RNAi*^ (VDRC: KK 108554), *UAS-PDH*^*RNAi*^ (VDRC: GD 40410), *UAS-Acon*^*RNAi*^ (BDSC: 34028), *UAS-Mdh2*^*RNAi*^ (BDSC: 36606), *UAS-Mpc1*^*RNAi*^ (VDRC: KK 103829), *UAS-PCB*^*RNAi*^ (VDRC: KK 105936), *UAS-kdn*^*RNAi*^ (BDSC: 36740), *UAS-ATPCL*^*RNAi*^ (VDRC: GD 30282), *UAS-Indy*^*RNAi*^ (VDRC: GD 9982), *UAS-tra2*^*RNAi*^ (BDSC: 28018), *UAS-AstA-R2*^*RNAi*^ (BDSC: 25935), *UAS-TkR99D*^*RNAi*^ (BDSC: 27513), *UAS-rk*^*RNAi*^ (BDSC: 31958), *UAS-Put*^*RNAi*^ (BDSC: 35195), *UAS-InR*^*RNAi*^ (BDSC: 35251), *UAS-LRP1*^*RNAi*^ (BDSC: 44579), *UAS-babo*^*RNAi*^ (BDSC: 25933), *UAS-LpR1*^*RNAi*^ (BDSC: 50737), *UAS-mgl*^*RNAi*^ (BDSC: 33940), *UAS-LpR2*^*RNAi*^ (BDSC: 54461), *UAS-tor*^*RNAi*^ (BDSC: 35639), *UAS-ITP*^*RNAi*^ (BDSC: 25799), *UAS-Tl*^*RNAi*^ (BDSC: 35628), *UAS-Mlx*^*RNAi*^ (VDRC: KK 110630), *UAS-Mondo*^*RNAi*^ (VDRC: KK 109821), *UAS-Met*^*RNAi*^ (VDRC: KK 100638), *UAS-Npc2e*^*RNAi*^ (VDRC: KK 100445), *UAS-NLaz*^*RNAi*^ (VDRC: KK 107553), *UAS-grnd*^*RNAi*^ (VDRC: KK 104538), *UAS-slif*^*RNAi*^ (VDRC: GD 45590), *UAS-AdipoR*^*RNAi*^ (VDRC: GD 40936), *UAS-EcR*^*RNAi*^ (VDRC: GD 37058), *UAS-usp*^*RNAi*^ (VDRC: GD 16893), *UAS-btl*^*RNAi*^ (BDSC: 60013), *UAS-gce*^*RNAi*^ (BDSC: 61852), *UAS-Mal-A1*^*RNAi*^ (VDRC: KK 106220), *UAS-CG6484*^*RNAi*^ (VDRC: KK 109484), *UAS-Pdp*^*RNAi*^ (VDRC: KK 107271), *UAS-Pdk*^*RNAi*^ (VDRC: KK 106641), *UAS-CG13907*^*RNAi*^ (VDRC: KK 107339), *UAS-Mct1*^*RNAi*^ (VDRC: KK 106773), *UAS-Prestin*^*RNAi*^ (VDRC: GD 5341), *UAS-Out*^*RNAi*^ (VDRC: GD 51157), *UAS-CG8925*^*RNAi*^ (VDRC: KK 101128).

##### Mutants

*tra*^*KO*^ (BDSC: 67412), *Df(3L)*^*st-j7*^ (BDSC: 5416), *tra*^*F K-IN*^ (constitutive *traF* knock-in, this study, see below for details), *Df(2R)*^*trix*^ (BDSC: 1896), *tra2*^*1*^ (gift from P. Schedl, Fujihara et al., 1978), *tra*^*FRT*^ (FRT-flanked *tra* knock-in, this study, see below for details), *snf*^*148*^ (BDSC: 7398), *esg*^*SHOF*^ (Voog et al., 2014), *zpg*^*z-2533*^ and *zpg*^*z-5352*^ (gift from Guy Tanentzapf, Arkov et al., 2006), *CCHa2-R*^*TAL34*^ and *CCHa2-R*^*KO51-2*^ (Sano et al., 2015), *Akh*^*AP*^ *and* A*kh*^*A*^ (Gáliková et al., 2015), *AkhR*^*1*^ (Grönke et al., 2007), and *Df(2L)*^*Exel7027*^ (BDSC: 7801).

### Method Details

#### FlyPAD assays

FlyPAD assays were performed as described in (Itskov et al., 2014). One well of the flyPAD arenas was filled with 2.4 μL of food (5% yeast 7% dextrose in 1% agarose) or our standard food, and the other was left empty. For all experiments, 5 day-old fed flies were individually transferred to flyPAD arenas by mouth aspiration and allowed to feed for 1-2hr at 25°C, 65% relative humidity. The total number of sips per animal over this period was acquired using the Bonsai framework (Lopes et al., 2015), and analyzed in MATLAB using previously described custom-written software (Itskov et al., 2014). Non-eating flies (defined as having fewer than two activity bouts during the assay) were excluded from the analysis. All flyPAD experiments were performed during the day from 11:00 until 15:00. N values shown in figures indicate the number of flies tested for each genotype. Data for experimental and control genotypes (or sexes) used for comparison was always acquired in the same flyPAD assay.

#### Immunohistochemistry

Intact guts were fixed at room temperature for 20 min in PBS, 3.7% formaldehyde. All subsequent incubations were done in PBS, 4% horse serum, 0.2% Triton X-100 at 4°C following standard protocols. To visualize the three-dimensional arrangement of the internal organs inside the male body cavity, intact abdomens were fixed at room temperature for 20 min in PBS, 3.7% formaldehyde prior dissection and cuticle removal.

The following primary antibodies were used: chicken anti-GFP (ab13970, Abcam) 1/10000, mouse anti-GFP (11814460001, Roche) 1/1000, chicken anti-beta Galactosidase (ab9361, Abcam) 1/200, rabbit anti-phospho-Histone H3 Ser10 (9701L, Cell Signaling Technology) 1/500, mouse anti-Fas3 (7G10, DSHB) 1/50, rabbit anti-Pyruvate dehydrogenase E1-alpha subunit (phospho S293) (ab92696, Abcam) 1/200, rabbit anti-Aconitase 2 (ab83528, Abcam) 1/200, rabbit anti-cleaved *Drosophila* Dcp-1 (Asp216) (9578S, Ozyme) 1/500, and rhodamine Phalloidin (R415, ThermoFisher scientific) 1/1000. Fluorescent secondary antibodies (FITC-, Cy3- and Cy5-conjugated) were obtained from Jackson Immunoresearch. Vectashield with DAPI (Vector Labs) was used to stain DNA.

#### Generation of Mal-A3^GFP^, Pgi^GFP^ and Amy-p^GFP^ transgenic reporter lines

The following GFP-tagged clones from the fosmid library TransgeneOme Resource (Source Bioscience (Sarov et al., 2016) were ordered for *Mal-A3*, *Pgi* and *Amy-p* respectively: CBGtg9060D0780D, CBGtg9060F0441D and CBGtg9060B10205D. The clones were sequence-verified and transgenic lines were established through ΦC-31 integrase mediated transformation (Bestgene). *attP* sites used were *VK33* (BDSC: 9750) for *Mal-A3* and *Amy-p*, and *attP40* (BDSC: 36304) for *Pgi*.

#### Generation of *Mal-A7-Gal4* driver

To generate a knock-in *Gal4* under the control of *Mal-A7* regulatory sequences, recombination mediated cassette exchange of the following insertion was performed: *Mi{y[+mDint2] = MIC}Mal-A7[MI00819]* (BDSC:32708). The swapping strategy previously described in (Diao et al., 2015) was employed. Briefly, the chromosome containing the MiMIC insertion (BDSC: 32708) was combined with a chromosome bearing a *Gal4* donor (BDSC: 603111). Flies with both components were then crossed to flies with both germline-expressing *Cre* and *ΦC-31* transgenes (BDSC: 60299). Offspring were then crossed to flies carrying a *UAS-GFP* reporter (BDSC: 60291) and the progeny were screened by fluorescence microscopy. Recombinants were selected to establish stable lines.

#### Generation of the excisable FRT-flanked *tra* knock-in allele (*tra*^*FRT*^)

To generate an excisable FRT-flanked *tra* knock-in allele, the *tra* locus (3869 nucleotides (nt) containing: *tra* coding region, the 1910 nt upstream and the 967 nt downstream) was cloned using the following primer pair: 5′-AAAACGGCCGGACAGCACAACCAGTTCCGAC-3′ and 5′-AAAACTCGAGATGCCCATCCCCTGCAATAC-3′. PCR was performed with Q5 high-fidelity polymerase from New England Biolabs (M0491S). The PCR product was digested with EagI and XhoI prior to cloning into the RIV FRTnMCS1FRT white vector (DGRC: 1333, generated by (Baena-Lopez et al., 2013). The construct was sequence-verified and a transgenic line was established through ΦC-31 integrase mediated transformation (Bestgene), using a recently generated amorphic allele of *tra* (Hudry et al., 2016) in which *tra* locus has been replaced by an *attP* site (BDSC: 67412). The generated allele rescue *tra* null mutant females to fertility.

#### Generation of the constitutive *traF* knock-in allele (*tra*^*K-IN*^)

To generate a constitutive *traF* knock-in allele, the *traF cDNA* (fused with the 353 nt upstream and the 310 nt downstream of *tra*) was cloned using the following primer pair: 5′-AAAAGAATTCAATTTGTTTTATTTGTGCCTG-3′ and 5′-AAAACTCGAGAGTTTCGTCCGCGGGTC-3′. PCR was performed with Q5 high-fidelity polymerase from New England Biolabs (M0491S). The PCR product was digested with EcoRI and XhoI prior to cloning into the RIV FRTnMCS1FRT white vector (DGRC: 1333, generated by (Baena-Lopez et al., 2013). The construct was sequence-verified and a transgenic line was established through ΦC-31 integrase mediated transformation (Bestgene), using a recently generated amorphic allele of *tra* in which *tra* locus has been replaced by an *attP* site (BDSC: 67412, generated by (Hudry et al., 2016). The generated allele behaves as constitutively feminising transgene and rescue *tra* null mutant females to fertility.

#### Generation of the *UAS-gRNAs* transgene for combined knockdown of digestive enzymes

To generate a UAS transgene carrying *gRNAs* targeting the *Mal-A1* (*gRNA:* AACTGCATCTATACGGAATCCGG), *Amy-p* (*gRNA*: TCTACAACATGGTGGCCTTCCGG) and *Amy-d* (*gRNA*: TCTACAACATGGTGGCCTTCCGG) genes, the three *gRNAs* were assembled from two overlapping PCR products. PCRs were performed with Q5 high-fidelity polymerase from New England Biolabs (M0491S). The final PCR product was then cloned into Bbs1 digested pCFD6 vector (Addgene: Plasmid #73915, generated by Port and Bullock, 2016). The construct was sequence-verified and a transgenic line was established through ΦC-31 integrase mediated transformation (Bestgene), using the VK05 (BDSC: 9725) *attP* site line.

#### Generation of *UAS* laconic sensor

The pcDNA3.1(-)Laconic plasmid (San Martín et al., 2013) was digested with BamHI and BclI. The resulting 2,254bp fragment was purified by electrophoresis and cloned into a pUAST vector previoulsy digested with BglII. Restriction enzyme analysis was used to confirm correct orientation of the insert. Transgenic fly strains were obtained by embryonic injection of the resulting *UAS-Laconic* vector (outsourced to Rainbow Transgenic Flies Inc, CA, USA). The expression efficiency of the recovered transformant lines was assessed by crossing them to a mushroom body *GAL4* driver. The line used in this study was the one found to have the highest expression and harbors an insertion into chromosome II.

#### Generation of *UAS* citrate sensors

Three different nanosensors for citrate were generated by gene synthesis (GenScript): CIT96, CIT8 and CIT0 (Ewald et al., 2011). CIT8 corresponds to the citrate binding domain of the *Klebsiella pneumoniae* histidine sensor kinase CitA (amino acids: 6-130), inserted between the FRET pair Venus/CFP; CIT96 carries a point mutation (K77A) decreasing the affinity for citrate and CIT0 is a control sensor which harbors a mutation (R66A) that completely abolishes citrate binding. These three sensors were cloned into the *pUASTattb* vector (PMID: 17360644) with EcoRI and NotI. The constructs were sequence-verified and transgenic lines were established through ΦC-31 integrase mediated transformation (Bestgene, attP site VK00028, DBSC: 9745).

#### RT-qPCR

RNAs were extracted from 20 whole flies using Trizol (Invitrogen). RNAs were cleaned using RNAeasy mini Kit (QIAGEN), and cDNAs were synthesized using the iScript cDNA synthesis kit (Bio-Rad) from 500 ng of total RNAs. Quantitative PCRs were performed by mixing cDNA samples (5 ng) with iTaq Universal SYBR® Green Supermix (Bio-Rad, #172-5124) and the relevant primers in 384-well plates. Expression abundance was calculated using a standard curve for each gene, and normalized to the expression of *Vha100-4*, which is not sexually dimorphic. For data display purposes, the average of the expression abundance was arbitrarily set at 100% for each gene for control males, and percentage of that expression is displayed for all sexes and genotypes. In graphs displaying expression of the five gut-specific sugar genes (*Amyrel*, *Mal-A1*, *Mal-A6*, *Mal-A7* and *Mal-A8*), the median of expression of these five genes taken together is also displayed for both sexes. See Table S3 for primer details such as sequences and efficiency.

#### RNA-seq

The RNA-seq transcriptional data of adult midguts obtained from virgin males, females, and *tra* mutant females used for Figures 2A and S1A is available from GEO under accession number GSE74775. A summary of relevant data for the intestinal sugar genes is provided in Table S1.

#### Metabolite measurements using FRET-based metabolite sensors

All imaging experiments were performed on dissected midguts or testes expressing laconic, the glucose sensor or the citrate sensors. Adult midguts or testes of 5-day-old flies were dissected in HL3 buffer (70 mM NaCl, 5 mM KCl, 1.5 mM CaCl_2_, 4 mM MgC_l2_, 10 mM NaHCO_3_, 115 mM sucrose, 5 mM trehalose, 5 mM HEPES; pH 7.1; around 350 mOsm). The dissected organs were placed into an open μ-slide (chambered coverslip, ibidi #80826) and analyzed using a confocal microscope. Fluorescent images were acquired using a 20x objective, and the following filter sets: excitation 405 nm, emission 470-522 nm (CFP channel); excitation 405 nm, emission 532-627 nm (FRET channel). For data analysis, regions of interest (ROI) were delimited and the average intensity of both mTFP and Venus channels over each ROI were calculated. The design of the laconic sensor is such that FRET from mTFP to Venus decreases when lactate concentration increases. To obtain a signal that positively correlates with lactate concentration, the inverse FRET ratio was calculated by dividing mTFP intensity by Venus intensity. For experiments with the FLII^12^Pglu-700μδ6 glucose sensor or the citrate sensors, the FRET ratio (YFP/CFP) was computed to obtain a signal positively correlated to glucose or citrate concentrations. For the experiments displayed in Figure 5, FRET efficiency was measured after acceptor photobleaching. Briefly, the fluorescence intensities of the donors before and after photodestruction of the acceptors were compared. For all sensors, increased fluorescence intensity of the donors (donor dequenching) was observed after bleaching of acceptors, indicating FRET occurrence.

#### GC-MS metabolomics of whole, dissected testes

Metabolite profiling analysis was performed by the metabolomics core of the University of Utah (http://cihd.cores.utah.edu/metabolomics/). Samples for GC-MS analysis were processed as previously described (Li and Tennessen, 2018). For each condition, four independent samples were collected from independent mating vials. Each sample is composed of 150 dissected testes from mated male flies.

All GC-MS analysis was performed with an Agilent 5977B GC-MS with HES source and an Agilent 7693A automatic liquid sampler. Dried samples were suspended in 40 μL of a 40 mg/mL solution of O-methoxylamine hydrochloride (MOX) in pyridine, and incubated for 1h at 30°C. 25 μL of this solution were added to auto sampler vials. 60μL of N-methyl-N-trimethylsilyltrifluoracetamide (MSTFA) were added automatically via the auto sampler and incubated for 30 min at 37°C with shaking. After incubation, 1 μL of the prepared sample was injected into the gas chromatograph inlet in the split mode with the inlet temperature held at 250°C. A 10:1 split ratio was used for analysis. The gas chromatograph had an initial temperature of 60°C for 1 min, followed by a 10°C/min ramp to 325°C and a hold time of 2 min. A 30 m Agilent Zorbax DB-5MS with 10 m Duraguard capillary column was employed for chromatographic separation. Helium was used as the carrier gas at a rate of 1 mL/min. Data was collected using MassHunter software (Agilent). Metabolites were identified and their peak area was recorded using MassHunter Quant. This data was transferred to an Excel spread sheet (Microsoft, Redmond WA). Metabolite identity was established using a combination of an in-house metabolite library developed using pure purchased standards, the NIST library and the Fiehn library. Data was normalized to both the sample mass and an internal standard (d27-myristic acid). Statistical analysis was performed using Metaboanalsyt 3.0 (http://www.metaboanalyst.ca/) (Xia and Wishart, 2016).

#### LC-MS metabolomics on adult hemolymph and whole fly

For hemolymph extractions, males were decapitated in groups of 15-20 and placed in a 0.5 ml Eppendorf tube perforated with a 30G needle. These Eppendorf tubes were placed inside 1.5 ml Eppendorf tubes and were centrifuged for 15 min at 1500 g at 4°C to collect their hemolymph as described in (Demontis and Perrimon, 2010). For each sample, hemolymph was pooled from a total of 120-280 mated males (final sample volume ranged from 3.5-11 μL). 3 samples were used per genotype. For whole flies, 8 samples of 5 mated males each were used for each genotype. 3 μL of each hemolymph sample were extracted with metabolite extraction solution (300 μL, 80% methanol, 0.1% formic acid (FA)), and whole fly samples were homogenized using a TissueLyser II (QIAGEN, Hilden, Germany) with a tungsten carbide bead (30 Hz, 3 min) in metabolite extraction solution (300 μL). Isotopically labeled citric acid (1,5,6-carboxyl-^13^C_3_ citric acid 99% - Cambridge Isotope Laboratories, USA) was added as an internal standard in the extraction solution (150 ng/mL). Following vortex mixing (30 s) and sonication on an ultrasonic water bath (10 min), samples were centrifuged (13,000 g, 10 min). Finally, the supernatants were collected, filtered using PTFE membrane (0.22 μm) and transferred to autosampler vials prior to injection on the liquid chromatography system. Total protein content was determined from the pellet obtained after centrifugation (haemolymph: protein precipitate; whole fly: tissue debris) by agitation in RIPA buffer (200 μL, 95°C, 1000 rpm, 10 min), centrifugation (13,000 g, 10 min) and measurement of protein content using a BCA assay kit (Pierce, Rockford, USA).

Chromatographic analyses were carried out on a Vanquish Flex Binary UHPLC system (Thermo Fisher Scientific Inc., MA, USA) coupled to a benchtop hybrid quadrupole-Orbitrap Q Exactive mass spectrometer (Thermo Fisher Scientific Inc., Bremen, Germany). Baseline separation of isocitric acid and citric acid was achieved using a C18 Accucore Thermo Scientific column (150 × 2.1 mm, 2.6 μm) equipped with vanguard column (30 × 2.1 mm, 2.6 μm), both held at a temperature of 40°C and a flow rate of 0.2 mL/min. Mobile phases were water with 0.5% formic acid (v/v) (Solvent A) and 90% acetonitrile with 0.5% formic acid (v/v) (Solvent B). The gradient elution was performed with a 0%–80% solvent B gradient over 5 min, followed by column washing and equilibration, yielding a total run time of 13 min. Ionization was performed in the negative ion mode using a heated electrospray ionization source (HESI), under the following conditions: spray voltage −3.0 KV, heater temperature 330°C, capillary temperature 320°C, S-lens RF level 50, sheath and auxiliary gas flow rate, 35 and 10 units, respectively. Mass accuracy was calibrated using a customised calibration solution prior to sample analysis. Data was acquired in profile mode using Parallel Reaction Monitoring (PRM) with information regarding all the compounds defined in the inclusion list (see Table below), at a MS2 resolution of 17,500 at m/z 200 and isolation window of m/z 2.0. Nitrogen was used as collision gas in the higher energy collision dissociation (HCD) cell with normalized collision energy (NCE) set to 10%. Automatic gain control (AGC) was set to 2e4 and maximum injection time 50 ms. Xcalibur version 4.1 was used for data acquisition and processing.

**Table:** 

Compound name	Mass (*m/z*)	Formula	Species	Retention time (min)	Start (min)	End (min)	NCE
Isocitric acid	191.01973	C_6_H_8_O_7_	[M-H]^−^	1.88	1.00	4.00	10
Citric acid	191.01973	C_6_H_8_O_7_	[M-H]^−^	2.35	1.00	4.00	10
Citric acid (1,5,6-carboxyl-^13^C_3_, 99%)	194.02979	[^13^C]_3_C_3_H_8_O_7_	[M-H]^−^	2.35	1.00	4.00	10

#### CE-MS metabolomics on adult hemolymph

Hemolymph was prepared as above. Each sample consisted of pooled hemolymph of a total of 120-280 mated males (final sample volume ranged from 6.4-10 μL), which was diluted 1:6 in distilled water prior to metabolomics analysis. Metabolome analysis was performed in 4 samples of fly adult body fluid per genotype using CE-TOFMS by Human Metabolome Technologies, Inc (HMT). Each sample was mixed with 450 μL of methanol containing internal standards (20 μM) and mixed. Then, chloroform (500 μL) and Milli-Q water (200 μL) were added, mixed thoroughly and centrifuged (2,300 g, 4°C, 5 min). The water layer (400 μL) was filtrated through a 5kDa cut-off filter (ULTRAFREE-MC-PLHCC, Human Metabolome Technologies, Yamagata, Japan) to remove macromolecules. The filtrate was centrifugally concentrated and resuspended in 50 μL of ultrapure water immediately before the measurement. The compounds were measured in the Cation and Anion modes of CE-TOFMS based metabolome as previously described (Soga et al., 2003). All CE−MS experiments were performed using an Agilent CE Capillary Electrophoresis System equipped with an air pressure pump, an Agilent 1100 series MSD mass spectrometer and an Agilent1100 series isocratic HPLC pump, a G1603A Agilent CE−MS adaptor kit and a G1607A Agilent CE−ESI−MS sprayer kit (Agilent Technologies). System control, data acquisition and MSD data evaluation were performed via a G2201AA Agilent ChemStation software for CE−MSD.

CE−MS Conditions for Cationic Metabolites. Separations were carried out on a fused silica capillary (50 μm i.d. × 100 cm total length) using 1M formic acid as the electrolyte. Sample was injected with a pressure injection of 50 mbar for 3 s (3 nL). The applied voltage was set at +30kV. The capillary temperature was maintained at 20°C using a thermostat and the sample tray was cooled below 5°C. 5 mM ammonium acetate in 50% (v/v) methanol−water was delivered as the sheath liquid at 10 μL/min. ESI−MS was conducted in the positive ion mode and the capillary voltage was set at 4000V. A flow of heated dry nitrogen gas (heater temperature of 300°C) was maintained at 10 L/min. In MS with selective ion monitoring (SIM), sets of 30 protonated [M+H]+ ions were analyzed successively to cover the whole range of m/z values from 70 through 1027.

CE−MS Conditions for Anionic Metabolites. A cationic polymer coated SMILE (+) capillary was obtained from Nacalai Tesque (Kyoto, Japan) and used as the separation capillary (50 μm i.d. × 100 cm total length). The electrolyte for the CE separation was 50 mM ammonium acetate solution, pH 8.5. Sample was injected with a pressure injection of 50mbar for 30 s (30nL). The applied voltage was set at −30 kV. ESI−MS was conducted in the negative ion mode and the capillary voltage was set at 3500 V. In MS with SIM, sets of 30 deprotonated [M−H]- ions were analyzed successively to cover the whole range of m/z values from 70 through 1027. Other conditions were the same as in cationic metabolite analysis.

Peaks detected in CE-TOFMS analysis were extracted using automatic integration software (MasterHands ver. 2.17.1.11 developed at Keio University) in order to obtain peak information including m/z, migration time (MT), and peak area. Putative metabolites were then assigned from HMT’s standard library and Known-Unknown peak library on the basis of m/z and MT. All the metabolite concentrations were calculated by normalizing the peak area of each metabolite with respect to the area of the internal standard and by using standard curves, which were obtained by single-point (100 μM or 50 μM) calibrations. Hierarchical cluster analysis (HCA) and principal component analysis (PCA) were performed by statistical analysis software (developed at HMT).

### Quantifications and Statistical Analyses

#### GFP and pH3 quantifications

Mitotic and meiotic indices were quantified by counting pH3-positive cells in > 40 testes or > 10 midguts per genotype and/or condition (e.g., male or female).

For quantification of intestinal GFP protein expression level, a midgut portion (corresponding to R2 or R4 regions) was imaged at 20x magnification. GFP level was quantified using ImageJ in areas of identical size across all genotypes. Threshold was adjusted for the GFP channel (ImageJ function: Image > Adjust > Threshold) to subtract background, then the size and the intensity mean of the area above the threshold was considered (ImageJ function: analyze particles). Data was collected from at least 10 midguts per genotype and/or sex, and is displayed as boxplots showing all data points.

For quantification of testis GFP protein expression patterns, whole testes were imaged at 20x magnification. GFP area was quantified using ImageJ. Threshold was adjusted for the GFP channel (ImageJ function: Image > Adjust > Threshold) to subtract background, then the size of the area above the threshold was considered (ImageJ function: analyze particles) and averaged by testis size. Data was collected from at least 25 testes per genotype, and is displayed as boxplots showing all data points.

#### Wing area measurements

Left wings of females and males were dissected, dehydrated in ethanol and mounted between slide and coverslip in Euparal mounting medium. Slides were dried on a heating block overnight (60°C). Wing areas were quantified using ImageJ by manually selecting the Cartesian coordinates of six landmarks that represent junctions of veins with the wing contour, and then measuring the number of pixels included in the resulting outline (method adapted from Trotta et al., 2007).

#### Statistics and data presentation

All statistical analyses were carried out in GraphPad Prism 7.04. Comparisons between two genotypes and/or conditions were analyzed with the Mann-Whitney-Wilcoxon rank sum test. The Mann-Whitney-Wilcoxon rank sum test does not require the assumption of normal distributions, so no methods were used to determine whether the data met such assumptions. All graphs were generated using GraphPad Prism 7.04. All confocal and bright field images belonging to the same experiment and displayed together in our figures were acquired using the exact same settings. For visualization purposes, level, and channel adjustments were applied using ImageJ to the confocal images shown in the figure panels (the same correction was applied to all images belonging to the same experiment), but all quantitative analyses were carried out on unadjusted raw images or maximum projections. In all figures, n denotes the number of midguts, wings, testes, flies or group of flies that were analyzed for each genotype. Data are presented as boxplots with all data points shown, p values from Mann-Whitney-Wilcoxon test (non-significant (ns): p > 0.05; ^∗^: 0.05 > p > 0.01; ^∗∗^: 0.01 > p > 0.001; ^∗∗∗^p < 0.001). Asterisks highlighting significant comparisons across sexes are displayed in gray boxes, whereas those highlighting significant comparisons within same-sex datasets are displayed in red boxes (for females) and blue boxes (for males).

### Data and Code Availability

The accession number for gene expression reported in this paper is GEO: GSE74775.

Data in this paper are available upon request to the Lead Contact.

## Supplemental Information

Table S1. Sex-Specific Expression of Intestinal Sugar Genes, Related to Figures 2A and S1 The first spreadsheet (“RNA-seq Hudry et al.”) shows raw abundance values for the intestinal sugar gene transcripts from female (F), male (M), and *tra* mutant masculinized midguts. Expression data were obtained from Hudry et al., (2016). The second spreadsheet (“FlyAtlas”) features relative male/female expression abundance ratios for intestinal sugar genes in brain/CNS, midgut and Malpighian tubules. Transcript expression data were obtained from FlyAtlas 2 (Leader et al., 2018).

Table S2. Metabolomics Data, Related to Figure 7 Each individual spreadsheet shows raw data for the different metabolomics experiments: targeted GC-MS quantification of multiple metabolites in testes of control versus *R2R4 > Indy-RNAi* males (related to Figure 7), targeted LC-MS quantification of citrate in adult male hemolymph of control versus *R2R4 > Indy-RNAi* males (related to Figure S7), targeted LC-MS quantification of citrate in the whole body of adult male controls versus *R2R4 > Indy-RNAi* males (related to Figure S7), and CE-MS profiling of adult male hemolymph of control versus *R2R4 > Indy-RNAi* males.

Table S3. Primers Used for RT-qPCR Experiments Related to STAR Methods

Table S4. Fly Stocks Used in This Study, Related to STAR Methods Sheet 2 lists all individual fly stocks used in this paper. Sheet 1 contains a list of full genotypes for all figure panels.
